# Steroid hormone levels vary with sex, aging, lifestyle, and genetics

**DOI:** 10.1126/sciadv.adu6094

**Published:** 2025-03-28

**Authors:** Léa G. Deltourbe, Jamie Sugrue, Elizabeth Maloney, Florian Dubois, Anthony Jaquaniello, Jacob Bergstedt, Etienne Patin, Lluis Quintana-Murci, Molly A. Ingersoll, Darragh Duffy, Milieu Intérieur Consortium

**Affiliations:** ^1^ Mucosal Inflammation and Immunity Team, Université Paris Cité, CNRS, Inserm, Institut Cochin, Paris 75014, and Department of Immunology, Institut Pasteur, Paris 75015, France.; ^2^ Translational Immunology Unit, Institut Pasteur, Université Paris Cité, Paris 75015, France.; ^3^ Frontiers of Innovation in Research and Education PhD Program, LPI Doctoral School, Université Paris Cité, Paris, France.; ^4^ Single Cell Biomarkers UTechS, Institut Pasteur, Université Paris Cité, Paris, France.; ^5^ Human Evolutionary Genetics Unit, Institut Pasteur, Université Paris Cité, CNRS UMR2000, Paris 75015, France.; ^6^ Institute of Environmental Medicine, Karolinska Institutet, Stockholm, Sweden.; ^7^ Department of Medical Epidemiology and Biostatistics, Karolinska Institutet, Stockholm, Sweden.; ^8^ Chair Human Genomics and Evolution, Collège de France, Paris, France.

## Abstract

Steroid hormone levels vary greatly among individuals, between sexes, with age, and across health and disease. What drives variance in steroid hormones and how they vary in individuals over time are not well studied. To address these questions, we measured 17 steroid hormones in a sex-balanced cohort of 949 healthy donors aged 20 to 69 years. We investigated associations between steroid levels and biological sex, age, clinical and demographic data, genetics, and plasma proteomics. Steroid hormone levels were strongly affected by sex and age, and a high number of lifestyle habits. Key observations were the broad impact of hormonal birth control in female donors and the relationship with smoking in male donors. In a 10-year follow-up study, we identified significant associations between steroid hormone levels and health status only in male donors. These observations highlight biological and lifestyle parameters affecting steroid hormones, and underlie the importance of considering sex, age, and potentially gendered behaviors in the treatment of hormone-related diseases.

## Teaser

Circulating steroid hormone levels are influenced by biological sex, age, lifestyle behaviors, and host genetics.

## INTRODUCTION

Steroid hormones are key biomolecules regulating diverse and complex developmental and physiological processes (*1*, *2*). Steroid functions include cell metabolism, immune cell behavior, resistance to stress, water regulation and salt balance, and development and maintenance of the reproductive system and secondary sexual characteristics. Steroid hormones can be separated into two groups and further divided into five subgroups. The first group, the corticoids, includes mineralocorticoids and glucocorticoids, and the second group, the sex steroids, is composed of progestogens, estrogens, and androgens. The corticoids are mainly produced by the adrenal glands (*1*, *2*). In male organisms, the testes are the primary source for androgen and progestogen synthesis, whereas in female organisms, estrogens, androgens, and progestogens are mainly produced by the ovaries. During pregnancy, the placenta can also make estrogens, androgens, and progestogens (*3*). In addition to these primary sources, the adrenal glands contribute to the synthesis of certain androgens and progestogens, and steroid hormones can also be synthetized by peripheral steroidogenesis in tissues, such as the brain, skin, thymus, adipose, and intestinal mucosa (*4*–*8*). Of note, peripheral steroidogenesis can change local hormone concentrations (*1*, *2*). Thus, steroid hormones have multiple sources depending on the needs and state of the organism.

Steroid hormone levels can vary greatly between the biological sexes and with age, playing an underestimated critical role in immune responses and disease risk and outcome (*9*–*11*). Sex, age, and reproductive cycle changes in steroid hormone levels are involved in many sex- and age-associated pathophysiologies and immune responses (*12*, *13*). For example, elevated cortisol causes Cushing’s syndrome, while Addison’s disease is a result of adrenal insufficiency and low cortisol levels (*14*, *15*). Both conditions are more prevalent in women, and peak incidence occurs between 30 and 50 years of age. Sex steroids, such as testosterone, affect asthma differentially in women and men over time, in that young boys are more susceptible than girls to this disease, but after puberty, women have a higher incidence (*16*). In certain pathologies, such as polycystic ovary syndrome, in which women have atypically high androgen levels, it is unclear whether the condition is the cause or the result of steroid hormone dysregulation (*12*, *17*). Dysregulation in steroid expression levels can affect the development of many diseases, such as rheumatoid arthritis or reproductive cancers, while others, such as sepsis or HIV, can cause steroid expression level dysregulation (*12*, *13*, *18*, *19*). Given their ability to induce or inhibit inflammation, steroid hormones, and, in particular, corticosteroids, are common therapies used in rheumatic diseases (*20*), asthma (*21*), and infection (*22*). Direct inhibition of sex steroids is also used as a therapy to treat malignancies, such as breast or prostate cancer (*23*). Overall, steroid levels are good indicators of health status; therefore, defining healthy ranges in females and males and understanding how they change over time may support new preventative and therapeutic approaches for a wide range of diseases.

Despite their clear role in homeostasis and immunity, studies of intra- and interindividual differences in steroid levels between females and males or across ages are notably limited. Many studies focus on one or a few steroids, without consideration of the impact of upstream or intermediate molecules and interconnected steroidogenesis pathways, or use techniques poorly adapted to measuring these molecules (*24*). To address this large gap in our knowledge, we measured the levels of 17 steroid hormones by targeted mass spectrometry (MS) in the Milieu Intérieur cohort of 949 healthy donors stratified for sex and age (half female and half male from 20 to 69 years of age) (*25*). We found that nearly all steroids exhibited sex- or age-related variation, that women taking hormonal birth control had profoundly lower estrogen, progestogen, and androgen levels as compared to those found in menopausal women, and that testosterone decreases may be specifically associated not only with aging but also with declining health status.

## RESULTS

### Steroid classes cluster distinctly between the sexes

To determine interindividual variation in a healthy human cohort, we measured the levels of 17 major steroid hormones, with representation of the five subgroups: 11-deoxycorticosterone, corticosterone, and aldosterone (mineralocorticoids); 11-deoxycortisol, cortisol, and cortisone (glucocorticoids); progesterone and 17α-hydroxyprogesterone (17-OHP) (progestogens); estrone (E_1_) and estradiol (E_2_) (estrogens); and dehydroepiandrosterone (DHEA), dehydroepiandrosterone sulfate (DHEAS), etiocholanolone, androstenedione, androsterone, testosterone, and dihydrotestosterone (DHT) (androgens), in the plasma of 1000 donors by liquid chromatography–MS/MS (LC-MS/MS). Fifty-one donors were excluded from downstream analysis because their sample could not be processed due to viscosity (7 donors) or they did not consent to make their data publicly available (44 donors), resulting in a final sample size of 949. Donors self-declared their gender in the initial recruitment visit (V0) (*26*). To specifically study biological sex as a variable and identify differences between female and male donors, we relied upon allosome (X and Y chromosomes) karyotype, identified by whole-genome single-nucleotide polymorphism (SNP) arrays (*26*).

**Fig. 1.**
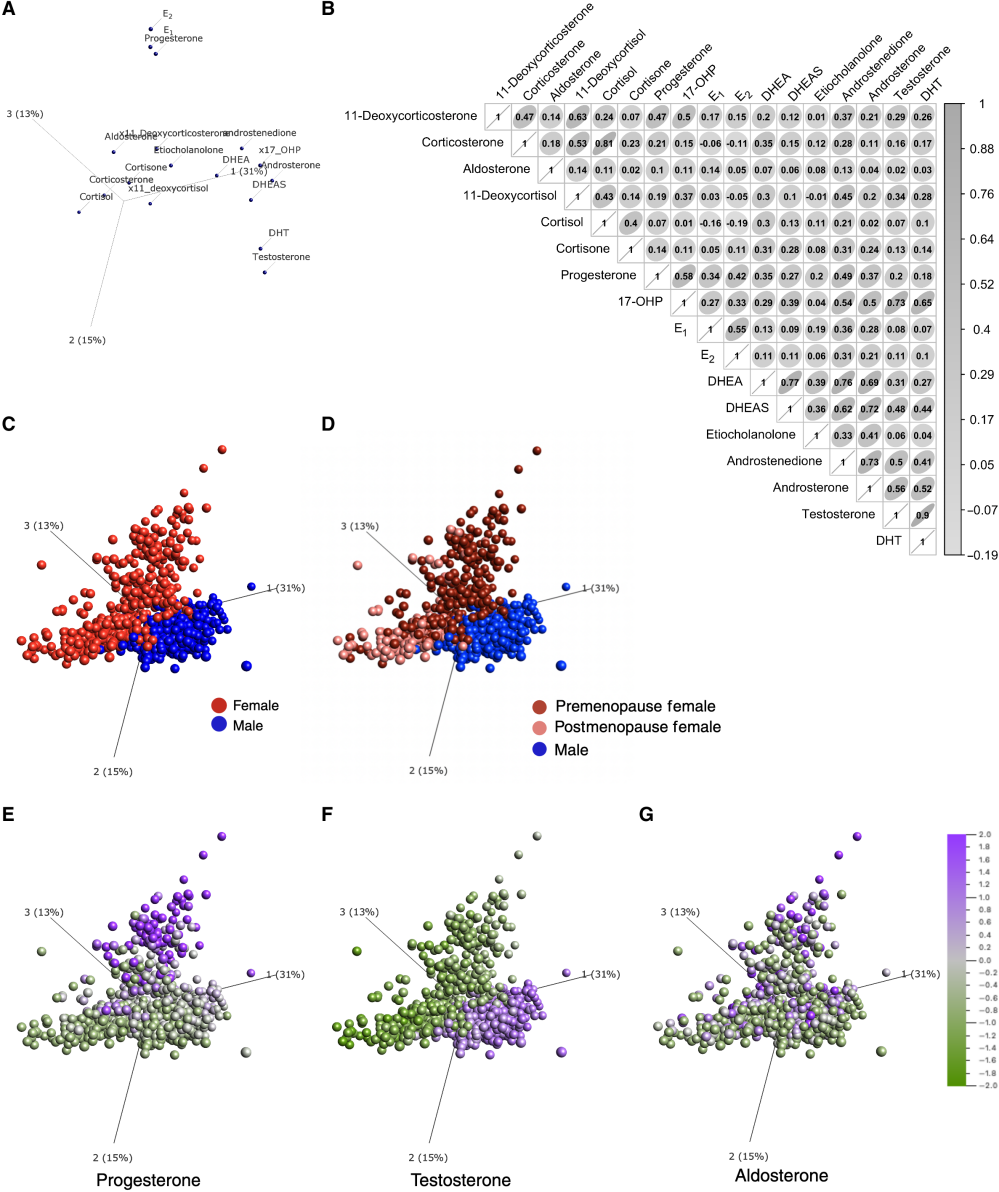
Steroid hormones and donors clustered by group and sex. We applied PCA to the log_2_-transformed nanomolar concentrations of steroid hormones measured by LC-MS/MS across 949 donors. (**A**) PCA shows clustering of steroid hormones across the first three PCs. (**B**) Correlation matrix shows Spearman correlations between hormones for all donors. Ellipses indicate the direction of correlation (to the right is a positive correlation). (**C** and **D**) PCA shows individual donors color-coded by (C) sex or (D) sex and menopausal status in female donors. (**E** to **G**) Expression levels of (E) progesterone, (F) testosterone, and (G) aldosterone overlaid on the PCA plot. [(A) and (C) to (G)] Proportion of variance explained by PC1, PC2, and PC3 are reported on the axes (*n* = 949 donors, 17 variables).

Applying principal components analysis (PCA) to reduce data dimensionality, we observed that steroid levels broadly clustered by group, in which the mineralocorticoids and glucocorticoids were closely clustered with each other (Fig. 1A). Progesterone and the estrogens clustered together, as did the androgens DHT and testosterone. The remaining androgens and the progestogen 17-OHP clustered together, distinct from the rest of the steroids. Pairwise correlation analysis showed that steroids that clustered the most closely together were the most correlated, such as the mineralocorticoid corticosterone and glucocorticoid cortisol (Fig. 1B). The androgens DHEA, DHEAS, androstenedione, and androsterone had strong positive correlations among each other and with 17-OHP. Given that testosterone is a precursor for DHT, and E_1_ and E_2_ can be converted into each other, their strong positive correlations were expected (figs. S1 and S2). Sex-specific correlation analysis showed differences in the strengths of the steroid relationships between female and male donors (fig. S2). For example, DHT correlated with both DHEA and DHEAS in female donors (*r*^2^ = 0.53 and 0.49, respectively) but not in male donors (*r*^2^ = 0.06 and −0.01, respectively) (fig. S2).

PC1, PC2, and PC3, which together explain 59% of the variance in steroid hormone levels, were strongly aligned with sex (Fig. 1, C and D). Progesterone and estrogen levels and DHT and testosterone levels drove sex-specific clustering of the donors (Fig. 1C), which could be further resolved by distinguishing pre- and postmenopausal women in the colored overlays (Fig. 1D). No donor was perimenopausal at the time of recruitment (*27*). Notably, sex steroids, such as progesterone (Fig. 1E) and testosterone (Fig. 1F), displayed the starkest differences between the sexes, whereas the mineralocorticoid aldosterone (Fig. 1G) had variable expression levels between the sexes and among pre- and postmenopausal women. The two corticoid groups had expression levels that were not strongly associated with female or male donors, and the remaining sex hormones showed the expected sex-specific patterns (fig. S3).

### Age affects steroid levels differently in female and male donors

**Fig. 2.**
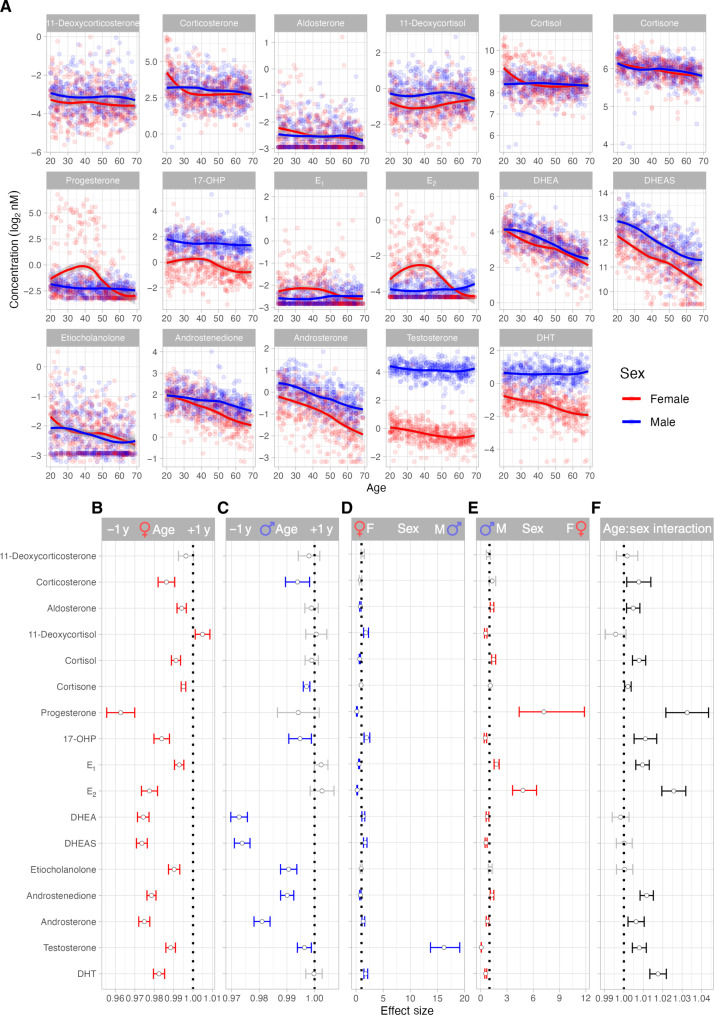
Steroid hormone levels differ with age and sex. (**A**) Scatterplots show log_2_-transformed steroid hormone nanomolar concentrations by age with applied LOESS models. Female donors are indicated in red, and male donors are indicated in blue. (**B** to **F**) Linear models were applied to quantify the effects of (B) age in female donors, (C) age in male donors, (D) sex, with female donors as the reference, (E) sex, with male donors as the reference, and (F) the interaction of age and sex on steroid hormone levels. FDR correction for multiple testing was applied globally for all three terms (age, sex, and age × sex interaction) over 17 steroid hormone variables. Significant effects (FDR *q* < 0.05) are indicated in red (female), blue (male), or black (both sexes), while nonsignificant effects are in gray (*n* = 949 donors).

To describe variability in individual steroid levels as a function of age and sex, we plotted log_2_-transformed nanomolar concentrations and applied locally estimated scatterplot smoothing (LOESS) models on the data (Fig. 2A). Untransformed steroid hormone concentrations are shown (fig. S4). To determine the strength of the relationships between steroid hormone levels and sex or age, we applied linear models to the log_2_-transformed nanomolar concentrations and calculated effect sizes on steroid levels, for age separately among female or male donors (Fig. 2, B and C), for sex (Fig. 2, D and E), and for age × sex interactions (Fig. 2F). To aid in interpreting the age × sex interactions, the effect sizes from the models applied with female donors as a reference (Fig. 2, B and D) and male donors as a reference are shown (Fig. 2, C and E). Notably, 12 of the 17 steroid hormones had a significant age × sex interaction [false discovery rate (FDR) *q* < 0.05] (Fig. 2F). Among the mineralocorticoids, 11-deoxycorticosterone did not change with age or between the sexes, whereas corticosterone and aldosterone levels declined with age, which was more pronounced in female donors (Fig. 2, A to C) as illustrated by the age × sex interaction (Fig. 2F). Levels of the glucocorticoids 11-deoxycortisol and cortisol were significantly different between the sexes. These differences were more pronounced in younger donors with a significant age effect in female donors only and an age × sex interaction effect on cortisol (Fig. 2, A, B, and D to F). Age was negatively correlated with cortisol and positively correlated with its precursor 11-deoxycortisol (Fig. 2B), suggesting age-related changes in cortisol biosynthesis. Cortisone levels were similar between female and male donors and declined with age, which was more pronounced in female donors as reflected in the significant age × sex interaction (Fig. 2, A to C and F).

From the mid-40s, progesterone levels dropped precipitously in female donors reflecting the proportional increase in menopausal donors from this age. By contrast, progesterone levels in male donors were low and relatively stable over time (Fig. 2, A to C). 17-OHP levels were significantly higher in male donors compared to female donors (Fig. 2, A, D, and E) and gradually declined with age in both sexes (Fig. 2, A to C). Estrogens E_1_ and E_2_ were higher in women and declined with age, whereas these hormones were less variable over time in men (Fig. 2). Last, the androgens DHEA, DHEAS, androstenedione, androsterone, testosterone, and DHT were higher in male donors than in female donors (Fig. 2, D and E). Etiocholanolone was not significantly different between female and male donors, potentially due to a high number of samples being at the limit of detection (LOD) (Fig. 2, A, D, and E). All the androgens measured significantly declined with age for both sexes, with the surprising exception of DHT, which did not decline in male donors, as reflected in the significant age × sex interaction (Fig. 2, A to C and F). We observed that testosterone levels were reduced in older donors (Fig. 2, E and F), but not as markedly as previous reports of testosterone decline in aging men, the so-called andropause (*12*).

### Lifestyle behaviors are markedly associated with steroid-level differences

**Fig. 3.**
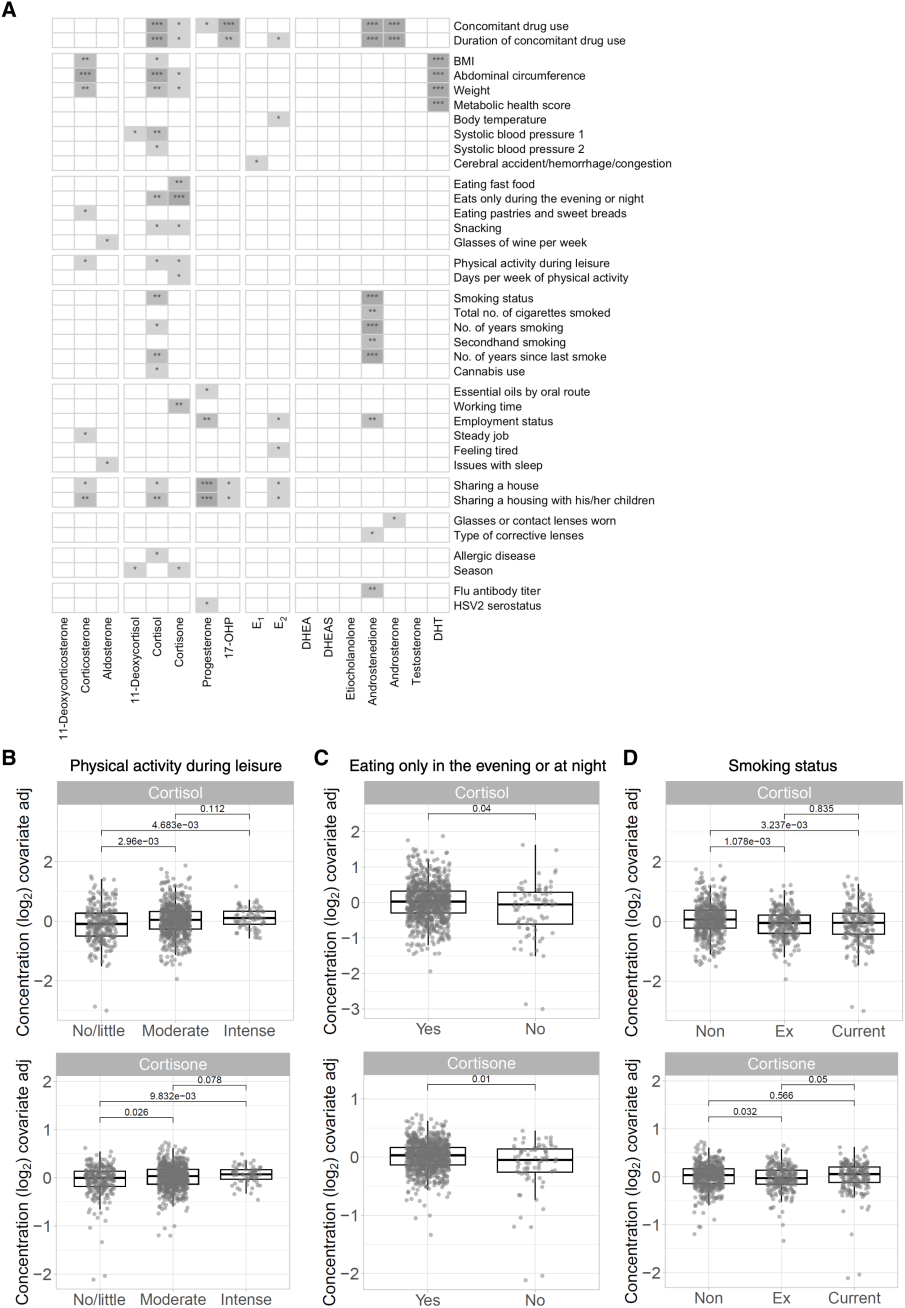
Specific characteristics and behaviors significantly associated with steroid hormone levels. (**A**) Heatmap of significant associations identified between steroid hormone levels and 137 CRF variables by LRTs with correction for sex, age, and sex-age interactions applying FDR correction (*q < 0.05, **q < 0.01, and ***q < 0.001). HSV2, herpes simplex virus 2. (**B** to **D**) Residual plots of the corrected values for cortisol and cortisone levels for (B) physical activity during leisure time (no/little, moderate, or intense physical activity), (C) eating only in the evening or at night (yes and no), and (D) smoking status (Non, nonsmoker; Ex, ex-smoker; Current, current smoker). *q* values were calculated using a Kruskal-Wallis with FDR correction for all tests together, followed by post hoc Dunn’s test (*n* = 949 donors).

We next determined whether specific lifestyle behaviors were associated with steroid levels while accounting for the expected influence of age and sex. We performed likelihood ratio tests (LRTs) for significant associations between the levels of each of the 17 steroids and 137 variables extracted from our study case report form (CRF) with all 949 donors. Because of the significant effect sizes found in the sex-age interaction analysis (Fig. 2F), we controlled regression models for age, sex, and the interaction between sex and age. Of 137 variables tested, we identified significant associations (FDR *q* < 0.05) with at least one steroid for 37 variables, in categories such as physiology, diet and alcohol consumption, physical activity and sleep, education and cohabitation, smoking, and medication, among others (Fig. 3A). Body mass index (BMI), weight, and abdominal circumference were associated with corticosterone and DHT levels (FDR *q* value for BMI = 6.376 × 10^–11^, for weight = 2.947 × 10^–6^, and for abdominal circumference = 6.005 × 10^–8^). DHT was also associated with a metabolic health score, which measures the cumulative risk for metabolic disease (*28*). The association of physiology-based categories with steroid levels was anticipated, as, for example, adipose tissue contributes to steroidogenesis (*29*).

However, we were surprised to observe associations between steroid hormone levels and specific behaviors, such as cortisone and eating habits (FDR *q* value for eating only at night = 3.019 × 10^–5^), fatigue and E_2_ (FDR *q* = 0.03), sharing a house and progestogens (FDR *q* < 0.01), smoking behavior and androstenedione levels (FDR *q* value for smoking status = 4.55 × 10^–8^), and concomitant medicine use and several glucocorticoids, progestogens, estrogens, and androgens (Fig. 3A). As the associations we identified were not always unidirectional, we plotted the residual values of cortisol and cortisone while controlling for sex, age, and the age × sex interaction, for physical activity during leisure, eating late, and smoking status to understand the nature of the associations between steroid levels and these lifestyle variables (Fig. 3, B to D). Cortisol and cortisone levels were significantly lower in donors who reported no to little physical activity compared to those who reported moderate or intense physical activity (Fig. 3B). Cortisol and cortisone levels were not different between donors who reported moderate physical activity and those who reported intense physical activity (Fig. 3B). Donors who reported frequent snacking between meals had lower levels of cortisol compared to those who never or sometimes snacked, whereas cortisone was only significantly lower in donors who reported snacking often versus never (Fig. 3C). Nonsmokers had significantly higher levels of cortisol compared to ex-smokers and current smokers, whereas cortisone levels were lower in ex-smokers versus nonsmokers (Fig. 3D).

As many of the variables in our CRF reflect or may be affected by socioeconomic status, we tested for associations between steroid hormone levels and socioeconomic status. To do so, we included in our LRTs a socioeconomic status score previously calculated using an Elo rating system (*30*). This score provides a relative ranking of the donors based on three socioeconomic status features: income, home ownership, and education, by performing multiple pairwise comparisons between donors. To avoid known age biases in income and home ownership, donors aged 20 to 29 years old were excluded from this specific analysis. We did not observe associations between steroid levels and socioeconomic status score, nor the individual factors, in our cohort.

### Lifestyle behaviors show sex- and gender-specific associations

**Fig. 4.**
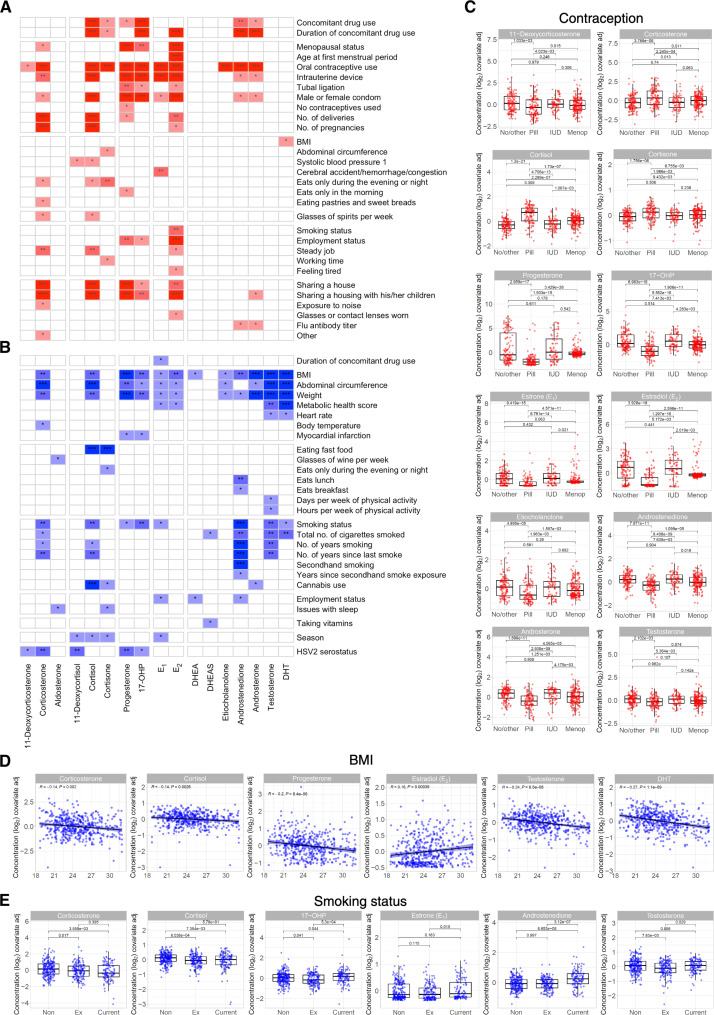
Sex-specific factors and gender-specific behaviors significantly associated with steroid hormone levels in women and men. (**A** and **B**) Heatmap of significant associations between steroid hormone levels and 151 or 137 CRF variables (number of variables tested in female or male donors, respectively) identified by LRTs of (A) female donors with correction for age, menopausal status, and the interaction between age and menopausal status or (B) male donors with correction for age, applying FDR correction (*q < 0.05, **q < 0.01, and ***q < 0.001). (**C** to **E**) Residual values were plotted for significant associations in (C) women for contraceptive usage (No/other, where other can be condom, tubal ligation; Pill, oral hormonal contraception; IUD, intrauterine device; Menop, postmenopausal women) and men for (D) BMI and (E) smoking status. *q* values were calculated using a Kruskal-Wallis test, followed by post hoc Dunn’s test and an FDR correction for all tests together [*n* = 472 (female donors) and *n* = 477 (male donors)].

As the LRT analysis of the entire cohort revealed specific associations with distinct sex steroids, we assessed whether specific lifestyle behaviors correlated with steroid levels differently between the sexes. As we previously did for the entire cohort, we performed LRTs for significant associations between the levels of each of the 17 steroids and variables extracted from the CRF, in only female or male donors separately. For the analyses of the female donors, we included 14 additional variables related to contraception, hormonal treatment, and history of pregnancies. The analysis was controlled for age for men and for age, menopausal status (pre- or postmenopausal), and the interaction between age and menopausal status for women (Fig. 4).

Of the 151 variables tested for women, we identified significant associations with at least one steroid for 30 factors, in the categories of contraception and medication, physiology, eating and working behaviors, and home ownership and living situation, among others (Fig. 4A). Significant associations were identified between several glucocorticoid and sex steroid hormones and the additional female-specific variables we included in the analysis such as menopausal status, age of menstrual period, number of pregnancies, and type of birth control methods (Fig. 4A). Analysis of the male donors identified 27 significantly associated variables of the 137 tested, in categories such as physiology, eating habits and activity level, and smoking habits (Fig. 4B). The significant associations we observed in the whole cohort analysis were not always present in both sexes or did not associate with as many steroid hormones. For example, diverse eating habits or smoking variables were significantly associated with steroid hormone levels only in men (Fig. 4, A and B). BMI, abdominal circumference, weight, and metabolic score were associated with 12 different steroid levels in men, but only cortisone and DHT were significantly associated with BMI and abdominal circumference in women (Fig. 4, A and B). Conversely and unexpectedly, sharing a home was only significantly associated with steroid levels in women. Whether the loss of certain significant associations was due to a loss of power (~500 donors of each sex versus ~1000 total donors) or these associations are driven by underlying sex or gender differences, such as those reported for smoking (*30*), will require further studies.

We observed that progesterone and the estrogens decline with age and were highly variable in premenopausal women (Fig. 2, A and B). This variability is likely influenced by both the menstrual cycle and hormonal contraceptive pill use. Notably, hormonal contraceptive pill use was significantly associated with 12 different steroid levels, including progesterone and the estrogens (Fig. 4A). We plotted these 12 steroid levels according to contraceptive pill use after adjusting for age, menopausal status, and the interaction between age and menopausal status (Fig. 4C). Quite notably, women using oral contraceptives had significantly lower levels of 11-deoxycorticosterone, a mineralocorticoid, as well as progesterone, 17-OHP, E_1_, E_2_, and the androgens etiocholanolone, androstenedione, and androsterone compared to both women not taking contraceptive pills and postmenopausal donors (Fig. 4C). In addition, among premenopausal donors, testosterone was significantly lower in those women using hormonal contraceptive pills compared to those not using this birth control method (Fig. 4C). Conversely, corticosterone, cortisol, and cortisone were significantly increased in women using hormonal contraceptive pills compared to donors not using contraceptive pills, including intrauterine devices (IUDs) and postmenopausal donors (Fig. 4C). Premenopausal women not using oral hormonal birth control had median steroid hormone levels more closely aligned with postmenopausal women than premenopausal women using oral contraceptives, likely reflecting the interrelatedness of steroidogenesis (fig. S1). These broad and significant changes in steroid levels were limited to women using oral hormonal birth control and were not apparent in women using other forms of contraception (Fig. 4C).

Two categories stood out for their broad associations with multiple steroid hormones in the analysis of male donors, BMI and smoking status. BMI, which in this cohort is within a healthy range (18.5 to 32 kg/m^2^) (*27*), was significantly associated with 12 of the 17 measured steroid hormones. We plotted residual values of 6 of the 12 most significantly associated steroid hormones (Fig. 4D). We observed that corticosterone, cortisol, progesterone, testosterone, and DHT levels were negatively correlated with BMI (Fig. 4D). E_2_ levels were positively correlated with BMI. Whether the correlations are causative remains to be determined. Plotting the residuals revealed different associations between steroid levels and smoking status (Fig. 4E). Corticosterone and cortisol concentrations were significantly lower in former and current smokers compared to nonsmokers (Fig. 4E). In addition, both E_1_ and androstenedione concentrations were significantly higher in current smokers than in former smokers and nonsmokers. There was no significant difference between nonsmokers and current smokers; however, 17-OHP and testosterone levels were lower in ex-smokers than in the two other groups.

### Common genetic variants influence steroid hormone levels

**Fig. 5.**
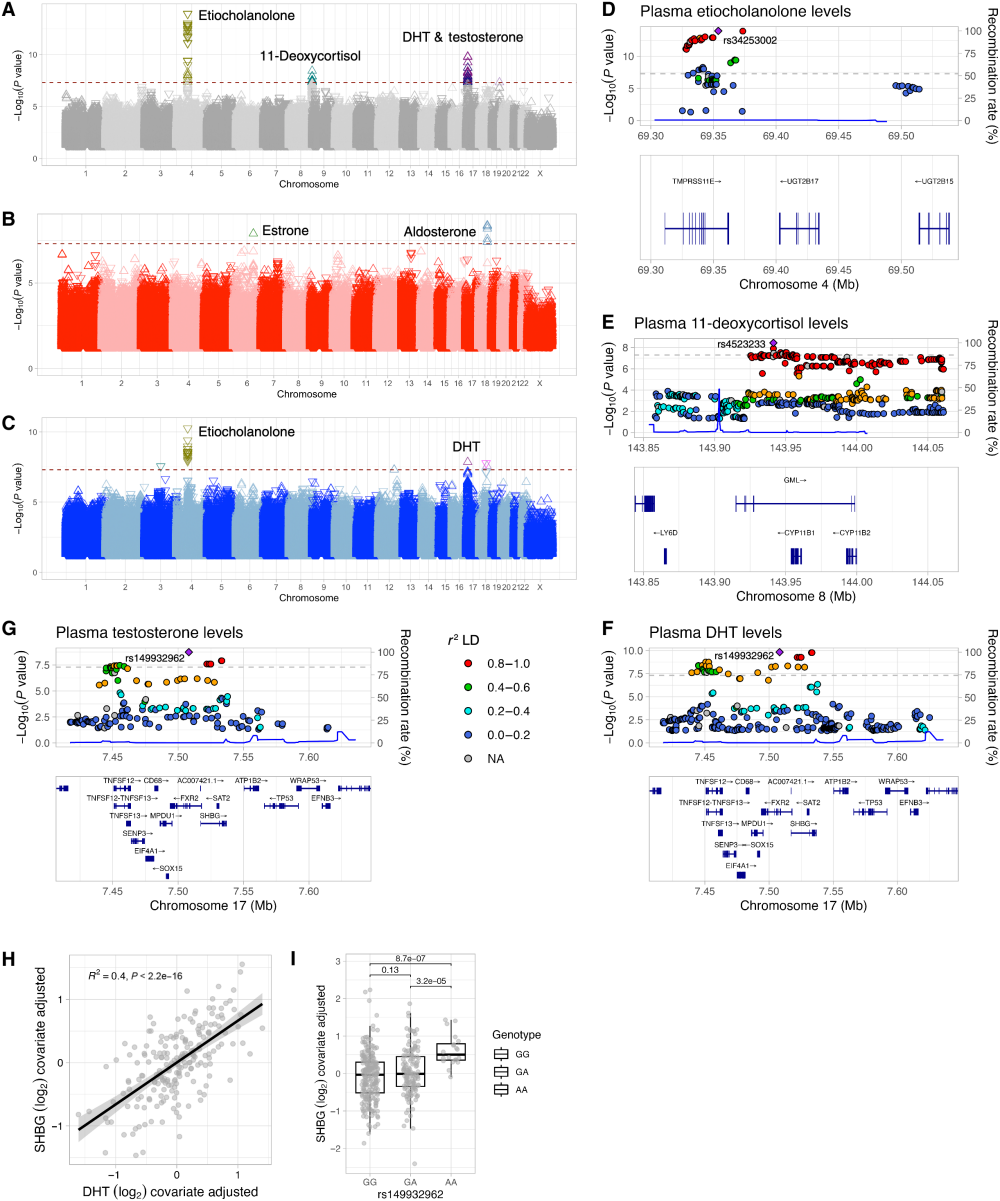
Steroid hormone levels are affected by genetic loci. (**A** to **C**) Summary Manhattan plots of associations between plasma steroid hormone levels and common genetic variants in the Milieu Intérieur cohort for (A) meta-analysis of results from female and male donors, (B) female donors, and (**D**) male donors. The red dotted line denotes a genome-wide significance threshold of *P* < 5 × 10^−8^. The direction of the triangles indicates the effect of the variant genotype on steroid levels. (C, **E**, and **F**) Locus zoom plots of SNPs associated with (C) etiocholanolone levels, (E) 11-deoxycortisol levels, and (F) DHT and (**G**) testosterone levels. The color of the locus plots denotes the level of linkage disequilibrium (*r*^2^), with the index SNP shown in purple. Only protein-coding genes are shown in the gene tracks. The blue line indicates recombination rates. NA, not applicable. (**H**) Residual plot showing correlation between DHT and SHBG. (**I**) Residual plot showing lead testosterone and DHT variant is a pQTL for SHBG levels in a subset of Milieu Intérieur cohort donors (*n* = 400). Data shown in the scatterplot (H) and box plot (I) are log_2_ data adjusted for covariates included in QTL analysis (age, BMI, smoking status, menopausal status, hormone replacement therapy, oral contraceptive use, IUD, and tubal ligation) and by sex. Pairwise comparisons were performed on adjusted data using Kruskal-Wallis tests followed by Dunn’s post hoc test with an FDR adjustment [*n* = 949 donors (all), *n* = 472 (female donors), and *n* = 477 (male donors)].

Having found age, sex, and lifestyle factors to be associated with variation in steroid hormone levels to different extents, we next tested whether common human genetic factors influenced the 17 steroid hormone levels measured in our cohort. To do so, we performed genome-wide association studies (GWASs) with 5,728,127 high-quality variants for which our cohort has been genotyped (*26*). To identify sex-specific genetic associations, we performed the analysis in female and male donors separately and then performed a meta-analysis on both sexes (Fig. 5, A to C). Covariates included in the model were age, BMI, smoking status, menopausal status, hormone replacement therapy, oral contraceptive use, IUD, and tubal ligation based on our analysis of the lifestyle data in the cohort and previous UK biobank hormone genetic association studies (*31*). To account for multiple testing, a *P* value of < 5 × 10^−8^ was considered as significant.

In the meta-analysis of female and male results, we identified associations with three loci on chromosomes 4, 9, and 17 with etiocholanolone, 11-deoxycortisol, and DHT and testosterone, respectively (Fig. 5A). Using locus zoom plots, we identified the most likely genes associated with each hormone. On chromosome 4, we identified *UGT2B17* variants associated with increased etiocholanolone levels (Fig. 5D and fig. S5). These variants are upstream of *UGT2B17*, an enzyme involved in the glucuronidation of C19 steroids such as etiocholanolone, androsterone, and testosterone (*32*). *UGT2B17* is highly polymorphic and encodes one of the most commonly deleted genes in the human genome (*33*). Using statistical fine mapping, we identified rs34253002 to be the most likely causal variant. By exploring the Genotype-Tissue Expression (GTEx) project, an expression quantitative trait locus (eQTL) database (*34*), we observed that variants such as rs2708699, which were associated with decreased levels of etiocholanolone, are associated with increased expression of *UGT2B17* (Fig. 5C). Variants associated with an increase in etiocholanolone levels, such as rs976002, are associated with decreased expression of *UGT2B17* in whole blood and liver in GTEx (*34*).

We also identified associations between 11-deoxycortisol and the *CYP11B1* locus on chromosome 8 (Fig. 5E). CYP11B1 catalyzes the 11β-hydroxylation of 11-deoxycortisol to cortisol (*35*). The SNPs identified were associated with increased levels of 11-deoxycortisol (fig. S6), suggesting reduced enzymatic activity or decreased expression of the enzyme. Fine mapping in a 1-Mb region centered on the significant SNPs identified rs4523233 as the likely causal variant. Single-cell data from GTEx indicate that rs4523233 is associated with increased expression of CYP11B1 in the adrenal gland (*34*).

Last, we found associations between the sex hormone binding globulin (*SHBG*) locus on chromosome 17 and testosterone and DHT levels (Fig. 5, F and G, and fig. S7). Fine mapping indicates that rs149932962 is the most likely causal variant associated with both DHT and testosterone. SHBG binds steroid hormones to facilitate the transport of their inactive bound forms in circulation. SHBG binds DHT with high affinity and testosterone and E_2_ with lower affinities. We previously measured SHBG levels in 400 individuals of the Milieu Intérieur cohort using Luminex multi-analyte arrays (*36*). After adjusting the DHT and SHBG data for covariates included in the GWAS, we found a significant positive correlation between DHT and SHBG levels (Fig. 5H). We also found that the lead SNP in SHBG (rs149932962) is a baseline protein QTL (pQTL) for SHBG levels (Fig. 5I).

In female donors specifically, we found additional associations with aldosterone levels and variants located on chromosome 18 (Fig. 5B). These signals are located near the *TCF4* gene. In addition, a single variant near *NKAIN2* on chromosome 6 was associated with E_1_ levels (Fig. 5B). NKAIN2 (Na^+^/K^+^ transporting adenosine triphosphatase–interacting protein 2) is involved in the maintenance of ionic gradients across cell membranes, although its precise function is not known. It is most highly expressed in the central nervous system and may function as a tumor suppressor gene. Mutated forms are found in several cancers, including prostate cancer (*37*). In male donors, only variants that were associated with decreased etiocholanolone levels were significant. In addition, a single variant (rs149932962) in SHBG was associated with DHT levels.

### Steroid hormones are associated with plasma proteins related to physiology and inflammation

**Fig. 6.**
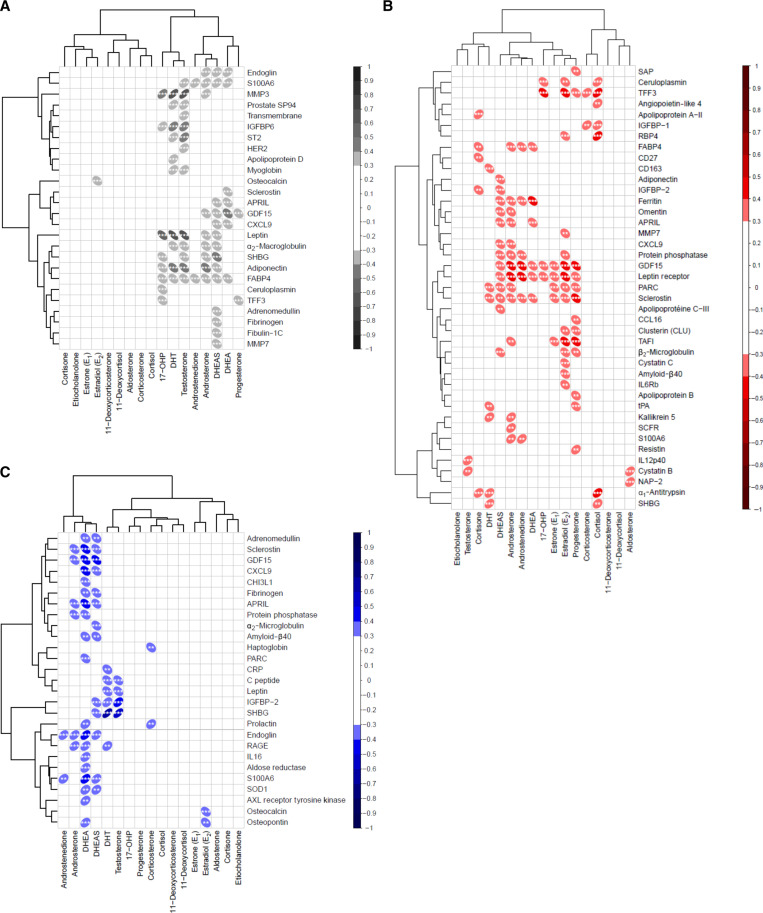
Plasma proteins have sex-specific associations with steroid hormone levels. (**A** to **C**) Heatmap of significant correlations between steroid hormone levels and 326 plasma soluble proteins from donors aged 30 to 39 and 60 to 69 (*n* = 371 donors) with Spearman correlation *R* > |0.3| of (A) all donors, (B) female donors, and (C) male donors, applying FDR correction (*q < 0.05, **q < 0.01, and ***q < 0.001). The direction of the oval indicates whether the relationship is positively (to the right) or negatively (to the left) correlated.

Given the identified associations between SHBG and DHT levels, we wanted to assess whether other plasma proteins were associated with specific steroid hormone levels. We performed correlation tests for significant associations between the levels of each of the 17 steroids and 326 soluble plasma proteins from donors aged 30 to 39 and 60 to 69 (*n* = 371 donors in total). The analysis was first performed for all donors together for maximum statistical power (Fig. 6A). To identify sex-specific associations, as observed in Fig. 4, the analysis was repeated separately for female (Fig. 6B) and male donors (Fig. 6C). Analysis of all donors identified significant associations between 27 proteins and at least one steroid hormone (Fig. 6A), many of which were found between plasma proteins and androgens in addition to progestogens, which clustered together. In addition, we observed two distinct clusters, in which 10 plasma proteins were significantly positively correlated with at least one steroid hormone and 17 were significantly negatively correlated with at least one steroid hormone.

In the sex-specific analyses, female-specific correlations were more numerous and observed with almost all the steroid hormones and a less defined clustering (Fig. 6B). SHBG was not significantly correlated with DHT levels when analyzing all donors together but was positively correlated with female and male donors when they were analyzed separately (Fig. 6, A to C). Correlations in the male-specific analysis were primarily with androgens (Fig. 6C). The only correlation with E_2_ concentrations was for osteopontin and osteocalcin in male donors. In addition, SHBG was positively correlated with cortisol in female donors and negatively correlated with DHEA and positively correlated with testosterone only in male donors (Fig. 6, B and C). Whether this finding is a sex-specific mechanism or due to limited statistical power is unclear.

### Steroid hormone level variations are explained by sex, age, genetics, physiological variables, and lifestyle

**Fig. 7.**
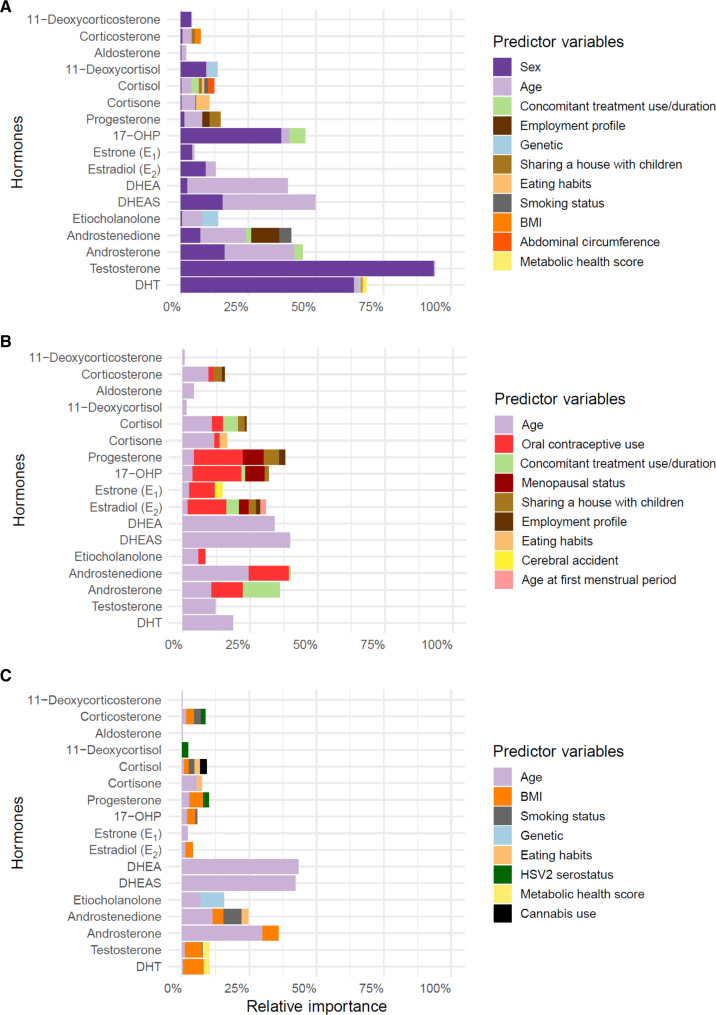
Sex and age explain a large proportion of interindividual variance in steroid hormone levels. (**A** to **C**) Graphs show the percentage of variance explained by each predictor for each hormone in (A) all donors (*n* = 949), (B) female donors (*n* = 472), or (C) male donors (*n* = 477). Average contribution of each predictor to the explained variance (*R*^2^) for each hormone is ordered by total importance across all hormones. Predictor variables with relative importance higher than 1% in at least one hormone are plotted.

To determine the relative contributions of different factors explaining interindividual variability in steroid hormone levels, we performed a relative importance analysis, including sex, age, significant clinical variables (*q* > 0.01), and the lead causal SNPs (identified by fine mapping) for each hormone in the entire cohort. While sex explained a large part of the variance for 17-OHP, testosterone, and DHT, and age explained variance in DHEA, DHEAS, androstenedione, and androsterone, much of the variance remained unexplained by the assessed parameters for the corticoids, estrogens, progesterone, and etiocholanolone (Fig. 7A). Compared to previously published studies for this cohort, in which sex explained on average 5% of variability in different immune phenotypes (*26*, *38*), the impact of sex and age on steroids was a major predictor variable. The same analysis was repeated independently for female and male donors to assess the sex-specific relative importance of the predictor variables (Fig. 7, B and C). We observed that while age still played an important role in sex-specific interindividual variability, oral contraceptive use had an impact on 10 of the 17 hormones measured (Fig. 7B). The use and duration of concomitant drug treatments and menopausal status also explained some of the observed variance in female donors. In male donors, BMI and smoking status were the main predictor variables for explaining interindividual variability in 9 of the 17 hormones (Fig. 7C).

### Steroid levels may reflect health and disease status

A feature of the original Milieu Intérieur study (*27*) was its cross-sectional design with a narrow inclusion period. The inclusion of only healthy donors deliberately restricted the heterogeneity of the cohort but, in doing so, limited extrapolation to disease development. To address this limitation, we recently re-recruited a subset (*n* = 415) of the original cohort 10 years after the initial study (*39*), for a third sampling visit (V3). All donors from the original study willing to participate in V3 were included, which allowed us to assess their health status 10 years after their original classification as healthy. Milieu Intérieur V3 participants were assigned a status of “healthy,” “at risk,” or “disease” determined by whether they would meet the original inclusion/exclusion criteria (healthy), they would be excluded on the basis of specific criteria (e.g., BMI) but were currently disease free (at risk), or they reported a disease at the time of the V3 study (disease) (*39*). Within these 415 donors, there was an age bias toward older age, but the equal sex balance was maintained (*39*).

**Fig. 8.**
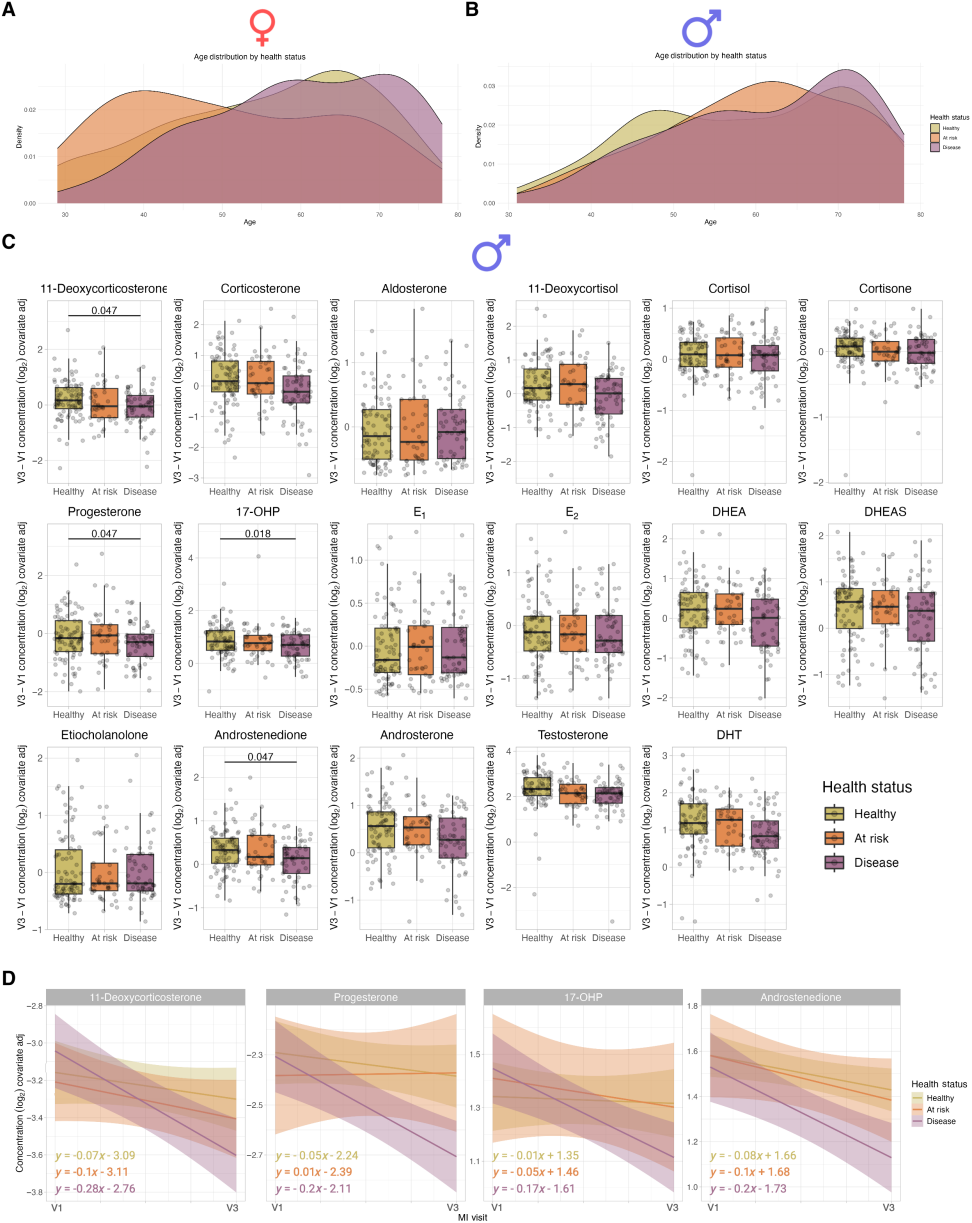
Declining steroid levels are significantly associated with a disease state only in men. (**A** and **B**) Ridge plot of the age distribution in each health group in (A) female donors and (B) male donors. (**C**) Box plots show the individual changes in covariate-corrected steroid hormone levels from V1 to V3 by health status in male donors. Significant *q* values are displayed from Mann-Whitney *U* tests comparing at-risk and disease groups to the healthy group, with FDR correction across all tests. (**D**) Regressions of covariate-corrected steroid levels in male donors from V1 to V3 by health group for steroids with significant Mann-Whitney *U* results (*n* = 204 male donors). MI, Milieu Intérieur.

To test associations between changes in steroid hormone levels over the 10-year period with health status, we measured steroid hormones from this time point (V3) together with the samples measured in Fig. 1 (V1) in a randomized fashion to avoid technical batch effects. Because of the age bias in the disease group (Fig. 8, A and B), we corrected all measures by applying linear models with age, circular time, and circular date to correct for circadian and seasonal steroid cycles, and used the residuals for subsequent analysis (*40*). To study the relationship between steroid hormone changes over time and health status, we calculated the individual differences between V1 and V3 residual measures, hypothesizing that the disease group would show a greater decline over the 10-year period. We did not identify any significant hormone changes associated with health status in female donors (fig. S8). In male donors, we observed significant differences between healthy and disease groups for 11-deoxycorticosterone, progesterone, 17-OHP, and androstenedione (Fig. 8C). We plotted the regression of 11-deoxycorticosterone, progesterone, 17-OHP, and androstenedione separately for the three groups over the 10-year period, which revealed the steepest decline in these hormones in the disease group as reflected by their slopes (Fig. 8D). The healthy group had the smallest decline in every hormone except progesterone, where the at-risk group showed a slight increase in concentration (positive slope) (Fig. 8D).

## DISCUSSION

This study describes diverse factors affecting steroid hormones in a well-defined healthy cohort balanced for sex and age, including a unique 10-year follow-up. Our analyses revealed expected findings, such as the decline of estrogens in older women compared to younger individuals. It also revealed unexpected findings such as the strong relationship between hormonal oral contraception and nonsex steroid hormones in women, and the widespread impact of BMI and smoking on steroid levels in men. It uncovered a relationship between specific steroid hormone decline over time and health status in men. A strength of the original Milieu Intérieur cohort is the robust health of all donors due to the stringent inclusion and exclusion criteria. This allows the study of older individuals in good health and provided a homogeneous initial health status for our comparisons in the 10-year follow-up study. From this analysis, it suggests that andropause and associated decline in testosterone in men may not be directly associated with aging, but rather with poor health. Another strength of our analyses is the ability to determine relationships between demographic data and steroid levels, although we cannot determine whether these are correlative or causative. Gender, economic and social status, and cultural habits influence behavior and lifestyle, with subsequent indirect effects on many biological processes (*30*). We identified that many of these behaviors are significantly associated with one or more steroid hormone levels. Some associations, such as housing or employment, may be due to nonlinear effects of age or other underlying factors, but are still important to consider in medical research.

Our study also has some important limitations. We have provided baseline sex- and age-specific ranges for steroid hormone levels in a cohort that is genetically homogeneous (i.e., European ancestry) and from a single geographical location. Thus, large-scale studies are needed on individuals with diverse genetic ancestries, geographical locations, and exposed to various environmental conditions (*41*). A second limitation is that we measured steroid hormone levels only in plasma. Circulating concentrations of steroid hormones may not fully reflect local concentrations in specific tissues, and in particular those that are capable of steroidogenesis (*4*–*7*). In addition, steroids can be found in high concentrations in other biological fluids, such as urine. Measuring steroid hormone concentrations in different tissues and biological fluids, and at multiple time points, would provide a deeper understanding of steroid hormone metabolism and the factors associated with it. Last, because of a large number of factors tested compared to our sample size, we reduced statistical power with each subdivision of the cohort (e.g., biological sex, menopausal, or health status) and when testing interaction effects. In addition, a large proportion of interindividual variance was not explained by variables measured in this cohort. This variance in steroid hormone levels is likely affected by many other factors, including specific enzymes that modulate hormones, such as aromatase and 5α reductase, which were not included in this study (*42*, *43*).

Many demographic factors were associated with corticosterone, cortisol, cortisone, or a combination of these steroids. These three glucocorticoids regulate stress, metabolism, and immunity (*1*, *2*). Cortisol has the highest activity, cortisone is the inactive form of cortisol, and corticosterone has a relatively lower activity serving as a precursor of aldosterone (*1*, *2*). The strongest associations were observed with physiological variables such as BMI or abdominal circumference in men, house sharing among women, and certain eating habits in a sex-specific manner. BMI and abdominal circumference were negatively correlated with cortisol in male donors, an interesting finding given that the inclusion criteria included a relatively narrow BMI range (≥18.5 and ≤32 kg/m^2^).

Quite notably, although oral hormonal contraception is commonly used, as exemplified by 40% of premenopausal donors in our cohort, the impact of oral hormonal contraception on the levels of many steroid hormones is not well understood. We observed that oral hormonal contraception use was significantly associated with 12 of the 17 steroid hormones measured. Younger donors not using this form of birth control and menopausal women had nonsex steroid levels that were more similar to each other than to levels in women using oral contraception. For example, premenopausal women using contraceptive pills had higher levels of cortisol and cortisone compared to premenopausal women not using this type of contraception, premenopausal women with IUDs, and postmenopausal women. High levels of cortisol are associated with weight gain, mental health disorders, increased cancer risk, cardiovascular diseases, and infection (*44*–*51*). Increases in cortisol levels may be directly or indirectly associated with some of the increased risk for certain types of cancer associated with oral contraception or even its impact on mental health (*52*–*56*). However, the use of oral contraception is also associated with a lower risk of ovarian cancer (*57*). In our study, the levels of progestogens, estrogens, and several androgens were lower in women using oral contraceptive pills than in postmenopausal women. These global alterations in steroid hormone levels in young women raise the question as to whether hormonal contraception, or other hormonal manipulation, affects health status concurrently or later in life and merits further study, given their widespread use. Hormonal contraception should also be considered in the context of general medical care.

Many of the smoking variables were associated with androstenedione levels, which were specifically higher in current smokers compared to past smokers or nonsmokers. Higher levels of androstenedione in smokers are described in different populations, although previous studies were performed separately in women (*58*–*60*) or men (*61*). While we only found this significant association in male donors, the proportion of female smokers was similar (19% female and 23% male donors). Alternatively, this lack of statistical significance could reflect other complex confounding factors, such as our previous observation that male smokers have lower socio-economic status compared to nonsmokers, which is not the case for female smokers (*30*). Smoking status was also associated with E_2_ levels in women, which is in line with a report that serum E_1_, E_2_, and estriol (E_3_) concentrations are lower in active and passive female smokers compared to nonsmokers (*62*). These differences were uncovered when we analyzed female and male donors separately, underlining the importance of studying both sex and gender effects. We recently reported that smoking has reversible impacts on innate immune parameters but long-lasting effects on adaptive immunity (*38*). Whether these impacts on immunity may be linked to effects on steroid hormone levels remains to be determined; however, smoking habits should be considered when treating hormone-associated diseases or using hormone-modifying treatments.

All steroid hormones showed a significant association with one or more plasma proteins measured, while six had significant genetic associations. These analyses are likely affected by the complex regulation of free and bound steroids in circulation supported by the strong associations revealed with SHBG, in both genetic and plasma protein analyses. Associations between etiocholanolone and variants upstream of UGT2B17, an enzyme involved in the glucuronidation of certain androgens, may be due to the metabolic elimination of this steroid. The addition of glucuronic acid from uridine 5′-diphosphate–glucuronic acid to etiocholanolone leads to increased water solubility and facilitates excretion in urine (*2*).

In summary, we present important insights into the causes of variability in steroid hormones in healthy women and men aged 20 to 79 years old. Despite some limitations, our work raises questions, such as the relationship between numerous steroid hormones and oral contraceptives, corticoids and activity levels or eating habits, or androgens and smoking behaviors in otherwise healthy individuals. Overall, sex, age, genetics, plasma protein levels, and lifestyle choices influence steroid hormone levels; however, how these complex relationships are mediated remains to be determined. These hypothesis-generating findings will support future mechanistic studies of steroid hormone levels and key immune functions in noninfectious, hormone-exacerbated diseases. Our data may serve as an important resource that will benefit the research community, not only in endocrinology and endocrine system–associated diseases but also in all fields in which steroid hormones play a role, including metabolism, immunology, and aging.

## MATERIALS AND METHODS

### Clinical protocol

Human samples were from the Milieu Intérieur cohort, which was approved by the Comité de Protection des Personnes-Ouest 6 on 13 June 2012 and by the French Agence Nationale de Sécurité du Médicament (ANSM) on 22 June 2012. The study was sponsored by Institut Pasteur (Pasteur ID-RCB number: 2012-A00238-35) and conducted as a single-center interventional study without an investigational product. The original protocol was registered under ClinicalTrials.gov (study no. NCT01699893). The samples and data used in this study were formally established as the Milieu Intérieur biocollection (NCT03905993), with approvals by the Comité de Protection des Personnes—Sud Méditerranée and the Commission Nationale de l’Informatique et des Libertés on 11 April 2018. Donors gave written informed consent. The 1000 donors of the Milieu Intérieur cohort were recruited by Biotrial (Rennes, France) and were composed of healthy individuals of the same genetic background (Western European) and to have 100 female donors and 100 male donors from each decade of life, between 20 and 69 years of age. Donors were selected on the basis of various inclusion and exclusion criteria that were previously described and self-declared their gender (*27*). To avoid the influence of hormonal fluctuations in female donors, pregnant and perimenopausal women were not included. The recruitment of donors was restricted to individuals whose parents and grandparents were born in metropolitan France and who had no family relationships, which minimizes genetic stratification.

The 10-year follow-up “V3” study was approved by the Comité de Protection des Personnes - Nord Ouest III (Committee for the protection of persons) on 27 January 2022 and by the French ANSM on 30 November 2011 (*39*). The study is sponsored by the Institut Pasteur and was conducted as a single-center study without any investigational product. The protocol is registered at ClinicalTrials.gov (study no. NCT05381857). As this study was a 10-year follow-up study of the original Milieu Intérieur “V1” study, the major inclusion criterion was previous inclusion in these studies. A health score labeled each V3 participant as healthy, at risk, or disease depending on the original Milieu Intérieur inclusion criteria: whether they would still be included, they would not pass inclusion, or they have a reported diagnosis, respectively. In addition, donors were required to give written informed consent and be affiliated with the French social security regimen.

### Steroid hormone measurements

For free steroid hormone measures, plasma samples from fasting EDTA-treated blood were frozen immediately upon collection at −80°C until aliquoting. Upon thawing, aliquoted samples were completely randomized before processing for MS analysis. Seventeen steroid hormones were measured using the AbsoluteIDQ Stero17 kit (Biocrates, Austria), which included glucocorticoids (11-deoxycortisol, cortisol, and cortisone), mineralocorticoids (11-deoxycorticosterone, corticosterone, and aldosterone), progestogens (17-OHP and progesterone), estrogens (E_1_ and E_2_), and androgens (androstenedione, androsterone, DHEA, DHEAS, DHT, etiocholanolone, and testosterone). Measurements of the 17 hormones were obtained from Biocrates in an Excel sheet along with a corresponding data report. Given that the measurements were taken across multiple plates, Biocrates calculated a project-based LOD, corresponding with the highest LOD in any plate for each steroid hormone. For measurements below LOD, Biocrates imputed missing values using the R package logspline. The logspline imputation method generates values between LOD and LOD/2 and applies the variance between values above LOD. Therefore, if there is a high variance in the dataset, then the imputed values may also exceed these limits.

### Datasets

#### CRF variables

Lifestyle, dietary, and medical history factors were collected in an electronic CRF-based questionnaire as previously described (*27*). To select factors for analysis, previously extracted and selected CRF variables (*38*) that had at least 5% of individuals in at least two categorical levels were used. The metabolic score, which varies from 0 to 1, was previously computed for each donor (*27*) by incrementing the score by 1 when abdominal circumference was >94 and >80 cm in male and female donors, respectively; systolic blood pressure was ≥130 mmHg or diastolic blood pressure was ≥85 mmHg; triglyceride levels were ≥1.7 mM; high-density lipoprotein levels were <1 and <1.3 mM in male and female donors, respectively; and glucose concentration was ≥6.1 mM (*28*).

### Genetic data

Protocols and quality control filters for genome-wide SNP genotyping were previously described (*26*). Briefly, the 1000 Milieu Intérieur donors were genotyped on both the HumanOmniExpress-24 and the HumanExome-12 BeadChips (Illumina, California, USA), which include 719,665 SNPs and 245,766 exonic SNPs, respectively. Variants that were unmapped on dbSNP138, duplicated, with low genotype clustering quality (GenTrain score of <0.35), with a call rate of <99%, or monomorphic were filtered out. For the X chromosome, SNPs that were heterozygous in >1% of male donors were removed. SNPs were further filtered for minor allele frequencies of >5%, yielding a dataset composed of 1000 donors and 5,715,171 SNPs for analysis.

### Plasma protein measures

Plasma proteins were previously described (*36*). Briefly, 326 soluble blood proteins were measured by multi-analyte Luminex assays (Rules Based Medicine) in the plasma of a subset of 400 donors.

### Data analysis

Unless otherwise stated, all displayed results were performed on the 949 individuals of the cohort who gave consent to share their data publicly. For analysis, the steroid hormone measures were log_2_ transformed. Raw concentrations are shown in fig. S4. Samples were prepared for shipments in two batches. Possible aliquoting batch effects were assessed using linear modeling. Batch effects were corrected using the removebatcheffect function from limma in R (version 4.2.2), where age and sex were included in the design parameter as biological variables that should be maintained. Data were log_2_ transformed in line with limma removeBatchEffect guidelines for the correction of the aliquoting batch. PCA plots were produced with Qlucore Omics Explorer (V3.9.9).

For visualization of steroid levels with age and sex, an LOESS function was applied with a span of 0.75 and a degree of 1. A linear model with the covariates of age, sex, and their interaction was applied to the steroid hormone levels. The subsequent estimate and corresponding confidence intervals were extracted from the models and exponentially transformed to report the effect sizes of these covariates. An age-sex interaction term was incorporated in all subsequent models.

### Electronic case report form (CRF) analysis

To test for associations between demographic variables and steroid hormone levels, we used LRTs comparing a base model controlling for age, sex, and an age × sex interaction [lm(hormone ~ age + sex + age:sex)] with a model controlling for age, sex, and an age × sex interaction plus a predictor demographic variable [lm(hormone ~ age + sex + age:sex + demographic variable)]. We performed this analysis on 137 demographic variables in all donors, with an additional 14 variables in female-only donors. An FDR correction was performed to account for multiple testing across all demographic features for each hormone. Heatmaps to display the *q* value from the LRT were generated using the R package pheatmap. For the residual plots, *q* values were calculated using a Kruskal-Wallis with FDR correction for all tests together, followed by post hoc Dunn’s test.

### Genetic association analysis

Genome-wide association testing was performed for each hormone using PLINK (version 1.9). Analysis was performed in female and male donors separately, and the results of both sexes were combined using meta-analysis. Covariates included for both sexes were age, BMI, and smoking status, as well as PC1 and PC2 of the genetic PCA to account for potential differences in population structure in the study cohort (*63*). Additional covariates included in the female-specific analysis included menopausal status, hormone replacement therapy, oral contraceptive use, IUD, and tubal ligation. Meta-analysis was used to combine the results of female and male donors for each hormone using PLINK (version 1.9). A *P* value of <5 × 10^−8^ was taken as significant. Quantile-quantile plots with λ values were used for quality control. Locus zoom plots were generated using the R package locuszoomr. Linkage disequilibrium for the plots was determined using data from the 1000 genomes project using the lead SNP identified by fine mapping using susieR as the reference. Manhattan plots were generated using ggplot2. Data for residual plots of individual SNPs were generated by running a linear model including all of the GWAS covariates using the tidy and augment functions from the R package broom. Male donors were considered as answering “no” for the female-only covariates for the purposes of this adjustment. Plots were generated using the ggpubr package.

### Fine mapping of genome-wide significant SNPs

Statistical fine mapping was performed on meta-analysis data using the susieR R package (version 0.12.35) in a 1-Mb window centered on the midpoint of significant SNPs within the same locus (*64*). *Z* scores were generated from summary statistics by dividing β values by the SE. Linkage disequilibrium matrices of *r* values were generated using PLINK v1.9 using Milieu Intérieur data. The susie_rss function was used, with the maximum number of credible sets (L) set to 10 and the number of iterations set to 100. For each locus, the SNP with the highest posterior inclusion probability (α) was selected as the index SNP.

### Longitudinal data

For comparison of steroid hormones over the 10-year sampling period, the log_2_-transformed concentrations were adjusted for age as well as circadian and annual rhythms by applying a linear model with age, and the sines and cosines of day of the year and time of day as the only covariates and extracting the subsequent residuals. Circular time and circular dates refer to a mathematical approach for representing cyclical variables, such as the time of day or day of the year, in a way that captures their continuous nature. We achieved this by transforming these variables into their sine and cosine components, which can then be included as predictors in a linear regression model [e.g., sin(π*time of sampling/24), cos(π*time of sampling/24), sin(π*day of sampling/365), and cos(π*day of sampling/365)]. This approach accounts for the fact that cyclical variables “wrap around”—for example, midnight is followed by 1 a.m., and December by January—ensuring that relationships in the data reflect their cyclical nature. The difference in adjusted steroid hormone concentrations over 10 years was calculated for each donor, then divided by sex, and grouped by health score. The adjusted hormone differences of the at-risk and disease groups tested against the reference healthy group via Mann-Whitney *U*, and subsequent *P* values were adjusted for multiple testing with FDR correction. An FDR *q* value less than or equal to 0.05 was considered significant.

### Computation of variance explained

For each steroid hormone, sex, age, the most significant clinical variables (*q* > 0.01), and the causal SNPs identified by fine mapping were considered to calculate the percentage of variance explained by each of these predictor variables with R package relaimpo 2.2.7 and plotted with the R package ggplot2 3.5.1. The *R*^2^ contribution averaged over orderings among variables was computed using the lmg type in the calc.relimp function of the relaimpo R package. For each hormone, genetic (rs34253002 for etiocholanolone, rs149932962 for testosterone and DHT, and rs4523233 for 11-deoxycortisol in all donors and rs138863991 for etiocholanolone in male donors), clinical variables such as eating habits (eating only at night or during the evening, eating fast food, and eating lunch), employment profile (employment status, working time per day, and steady job), and all the other most significant clinical variables (*q* < 0.01) per hormone were considered in the model.

## Supplementary Materials

### This PDF file includes:

Figs. S1 to S8 Millieu Intérieur Consortium author list
