# Supplementary material for: Steroid hormone levels vary with sex, aging, lifestyle, and genetics
Source: Sci Adv. 2025 Mar 28;11(13):eadu6094. doi: 10.1126/sciadv.adu6094 (PMC11952096; doi:10.1126/sciadv.adu6094)
Supplement: Supplementary file 1 — Figs. S1 to S8 Millieu Intérieur Consortium author list [file sciadv.adu6094_sm.pdf]

Science Advances
AAAS

Supplementary Materials for

Steroid hormone levels vary with sex, aging, lifestyle, and genetics

Léa G. Deltourbe *et al.*

Corresponding author: Molly A. Ingersoll, molly.ingersoll@pasteur.fr; Darragh Duffy, darragh.duffy@pasteur.fr

Sci. Adv. **11**, eadu6094 (2025)
DOI: 10.1126/sciadv.adu6094

This PDF file includes:

Figs. S1 to S8
Millieu Intérieur Consortium author list

Supplementary Figures

Supplementary Figure 1

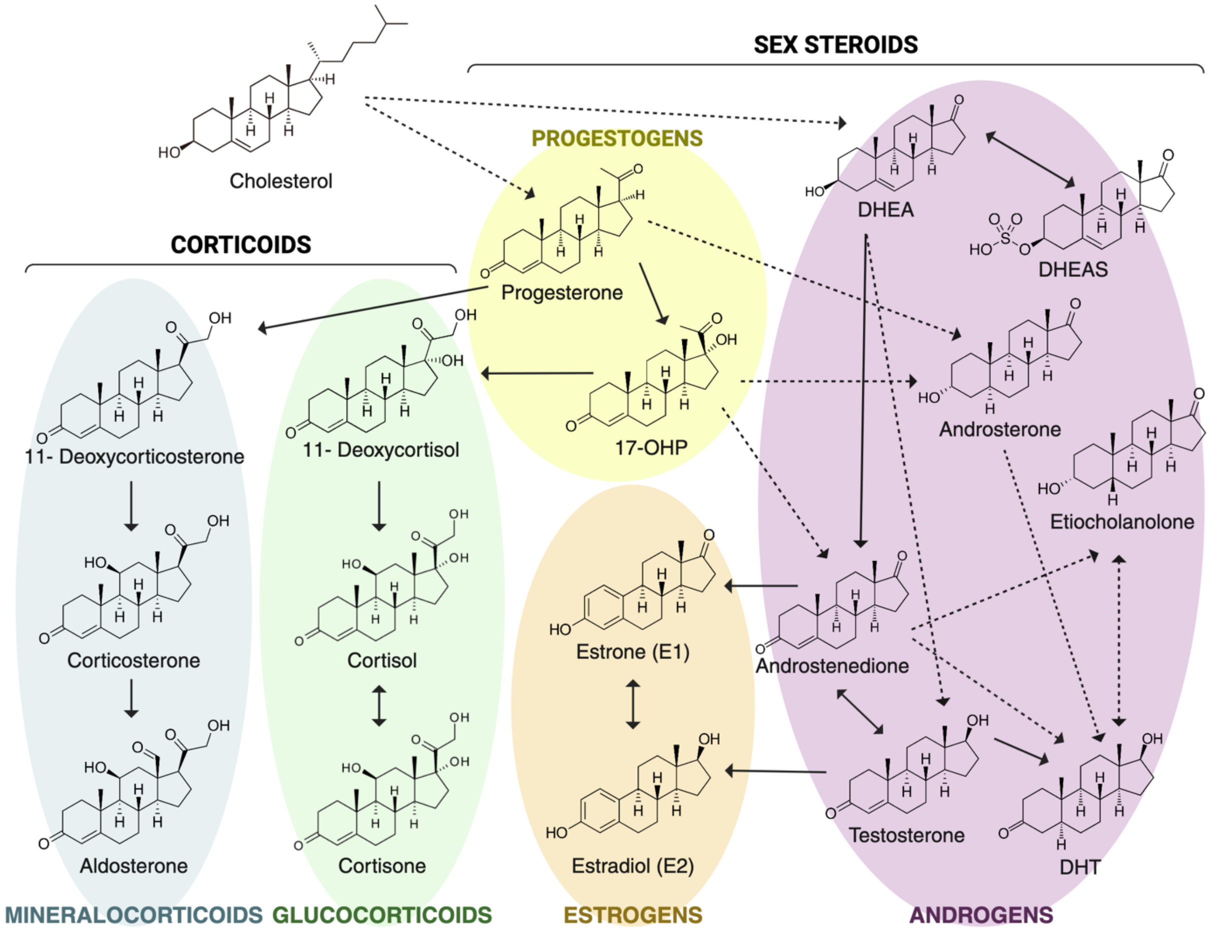

Supplementary Figure 1: Steroid hormones are interconnected in biochemical steroidogenesis pathways. Schematic overview of steroidogenesis and the relationship among the steroid hormones measured in this study[1,41,43]. Steroids are grouped into 2 categories: corticoids and sex steroids, which are then separated into 5 groups: mineralocorticoids, glucocorticoids, progestogens, estrogens, and androgens. Arrows show direct relationships between steroids, such as conversion by one enzyme. Dotted line arrows show relationships with at least one intermediate and two enzymes between the two steroids shown. 17-OHP: 17-hydroxyprogesterone, DHT: dihydrotestosterone, DHEA: dehydroepiandrosterone, DHEAS: dehydroepiandrosterone sulfate.

Supplementary Figure 2

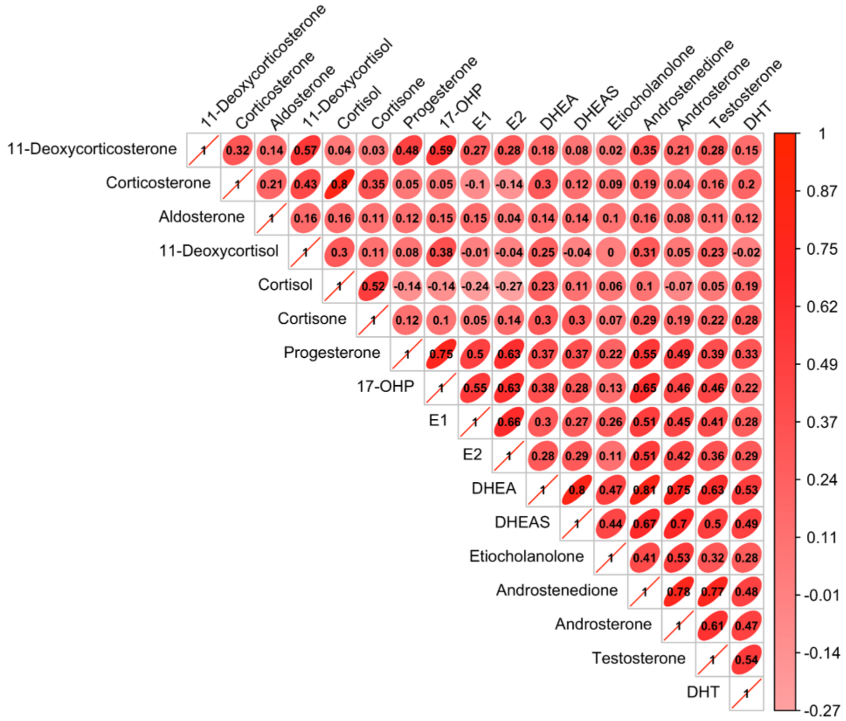

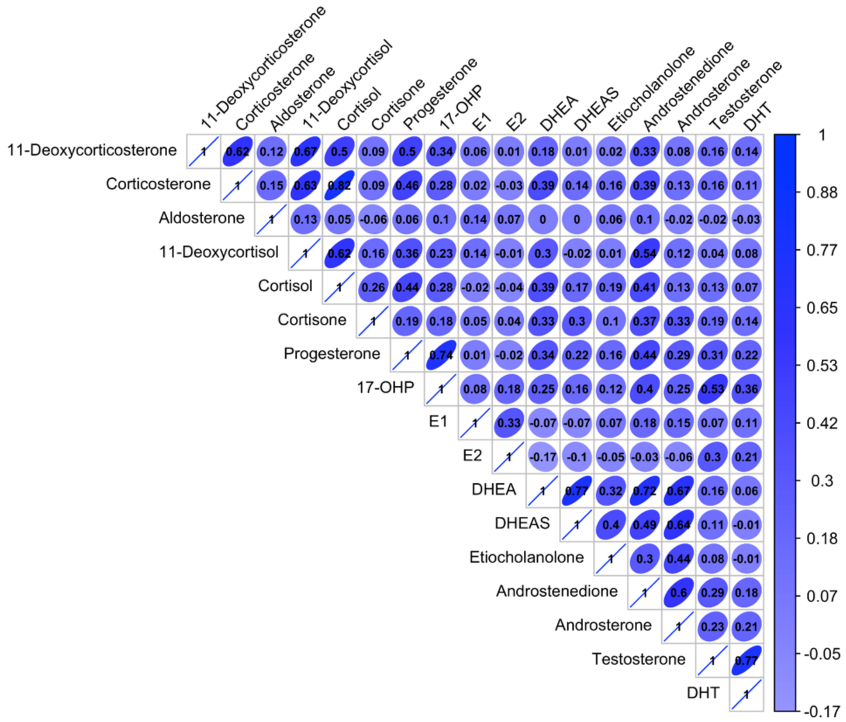

Supplementary Figure 2: Correlations between steroid hormone levels separately for female and male donors. Correlation matrices showing Spearman correlation between hormones using $\log_2$ data without adjusting for age separately for female (red) and male (blue) donors. r^2 values are displayed on the plot. Ellipses indicate the direction of correlation. n=472 females, n=477 males.

Supplementary Figure 3

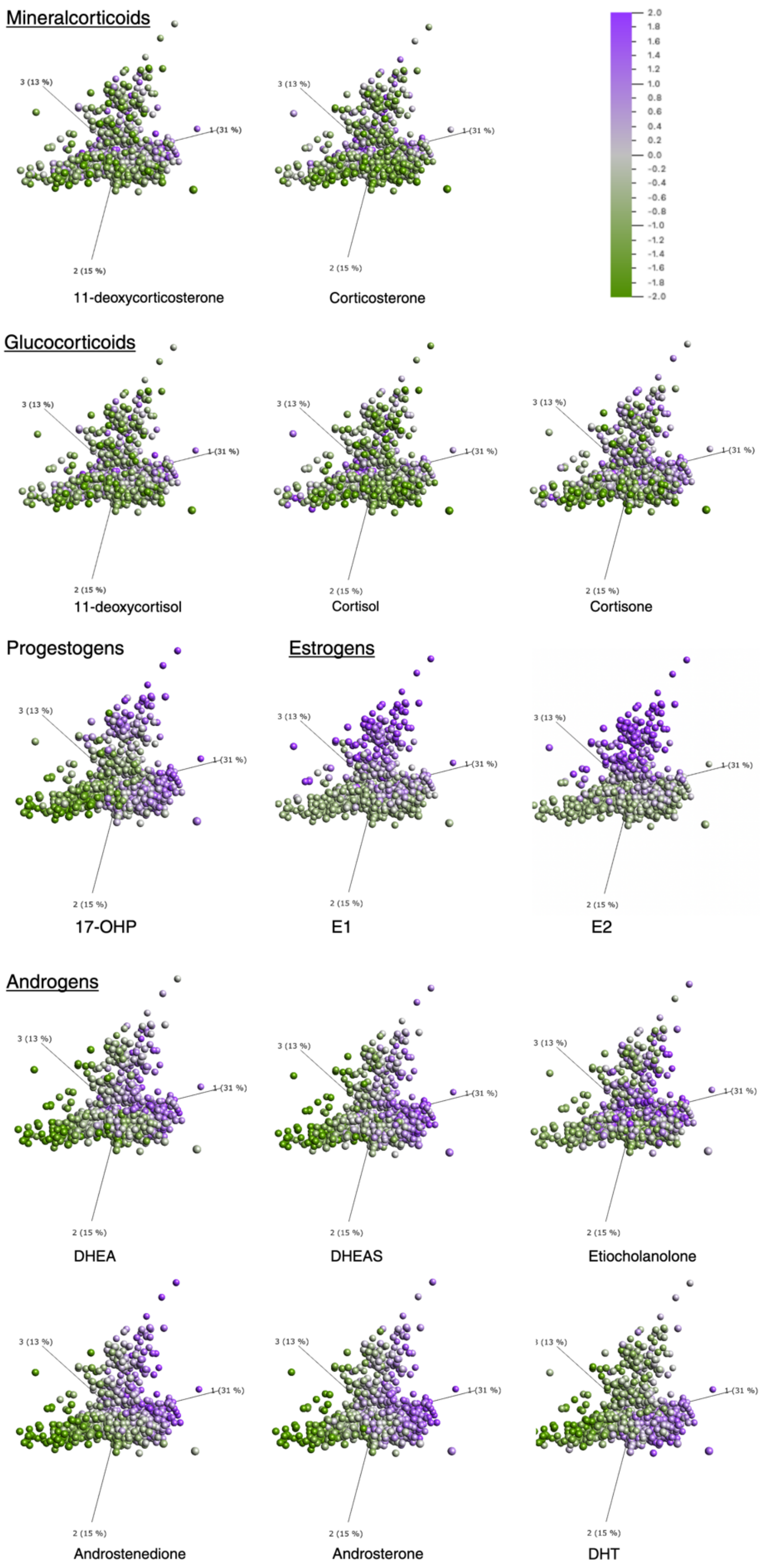

Supplementary Figure 3: Relative steroid hormone levels among a cohort of healthy donors. PCA of log-transformed nanomolar concentrations of steroid hormones measured by LC-MS/MS color coded by relative expression levels per hormone. (n=949 donors)

Supplementary Figure 4:

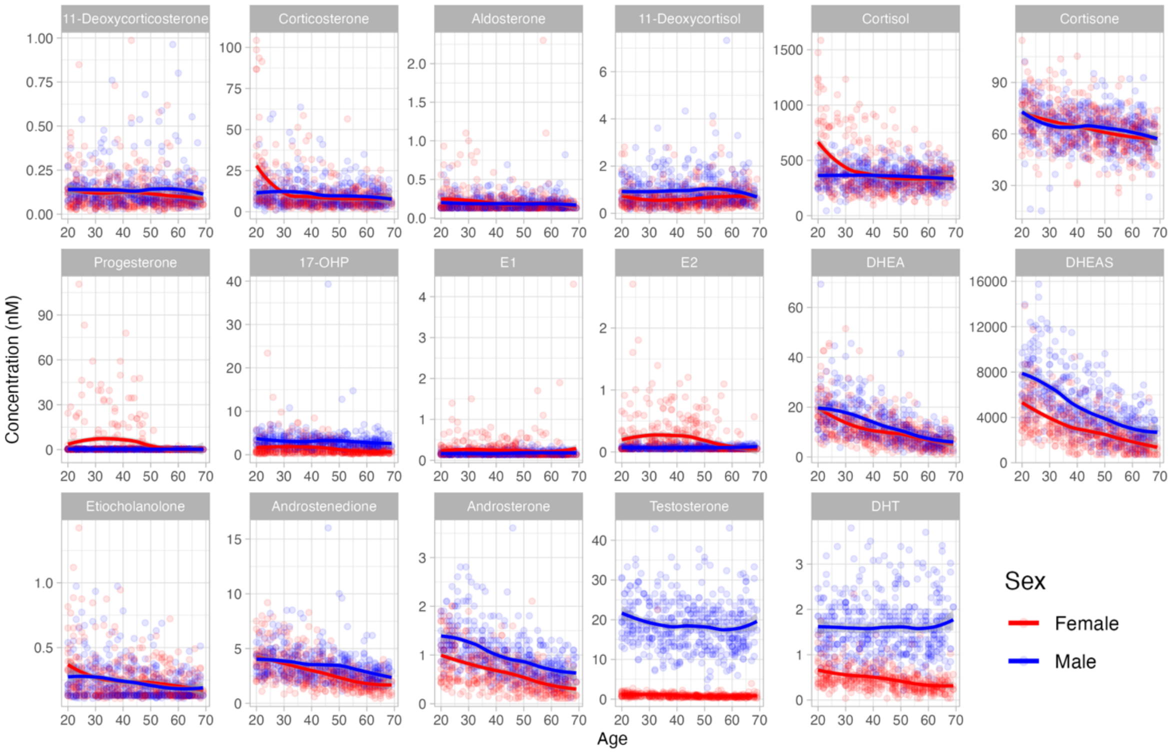

Supplementary Figure 4: Untransformed steroid hormone concentrations differ with age and sex. Scatter plots show untransformed steroid hormone nanomolar concentrations by age with applied LOESS models. Female donors are indicated in red, male donors in blue.

Supplementary Figure 5:

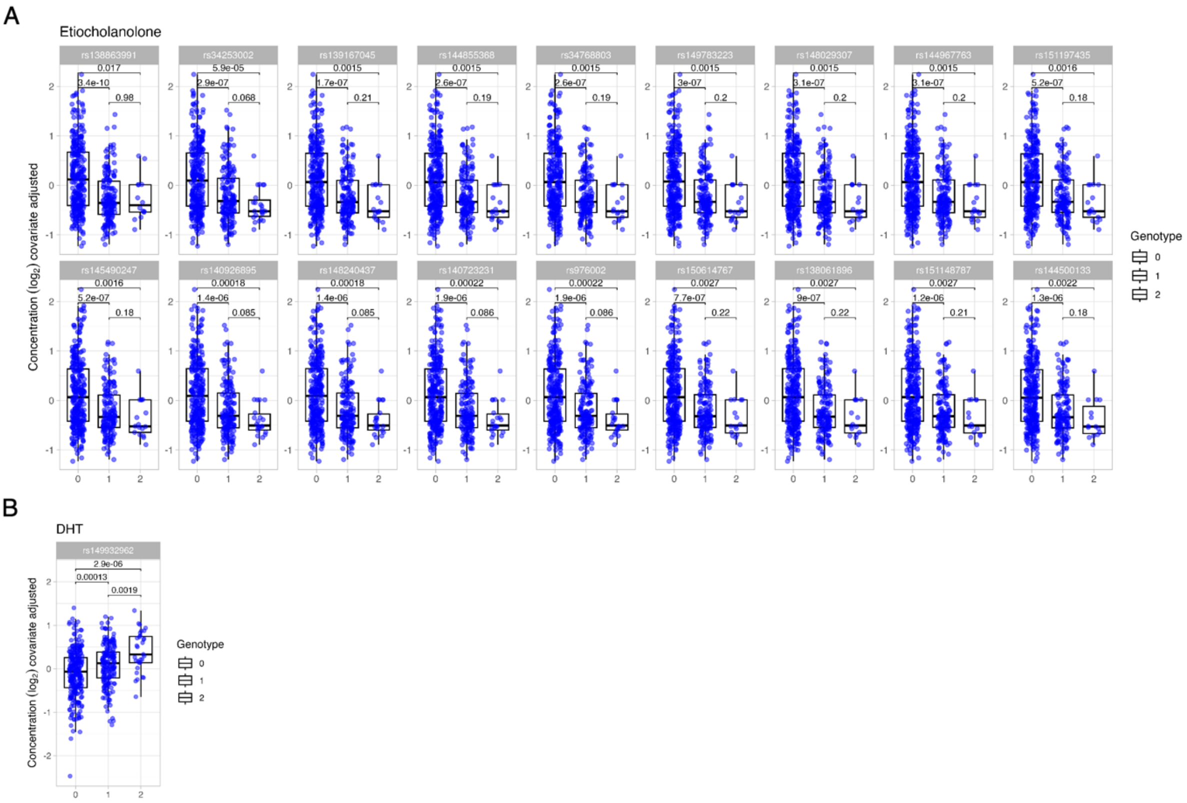

Supplementary Figure 5: SNPs associated with etiocholanolone and 11-deoxycortisol levels in male donors. Box plots of (A) etiocholanolone and (B) DHT levels in male donors for significant SNPs identified in the GWAS. Data has been corrected for covariates included in the GWAS (age, BMI, smoking status, as well as PC1 and PC2 of a genetic PCA, menopausal status, HRT, oral contraceptive use, IUD, and tubal ligation). P values displayed here are from a Dunn's test with an FDR adjustment. (n=477)

Supplementary Figure 6:

A Etiocholanolone

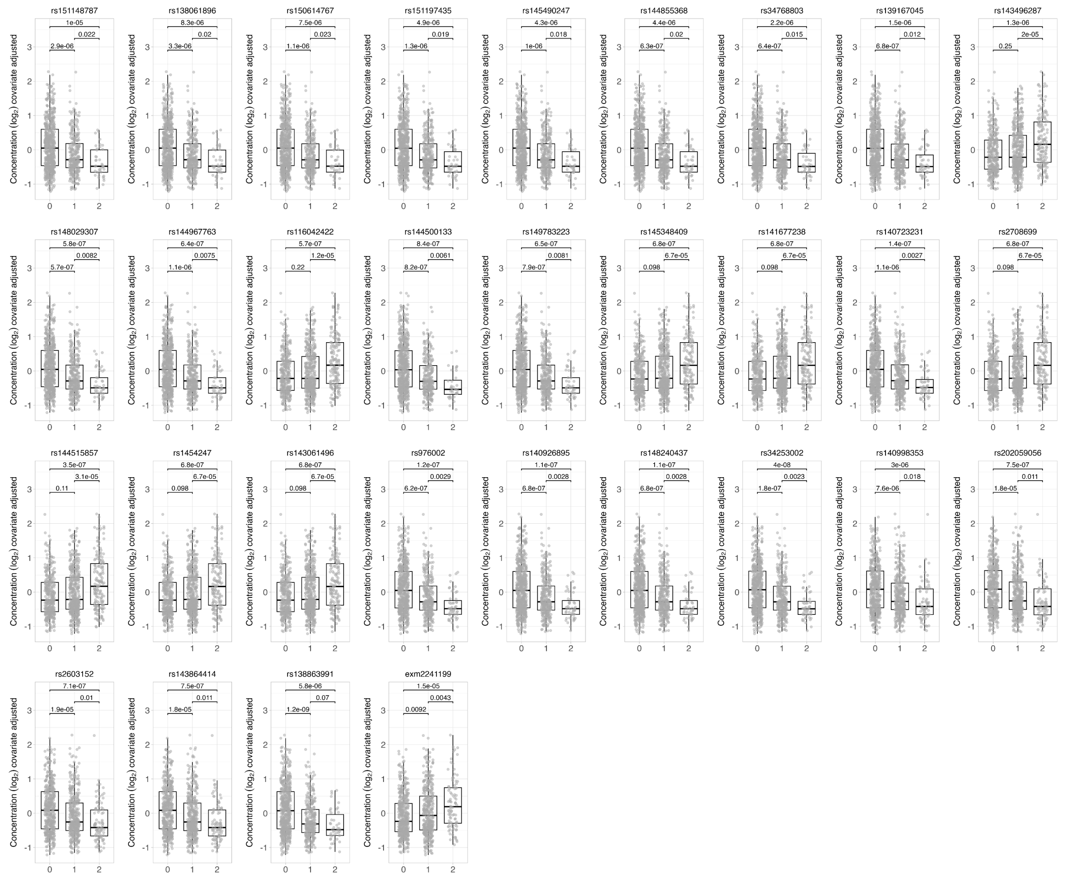

B 11-Deoxycortisol

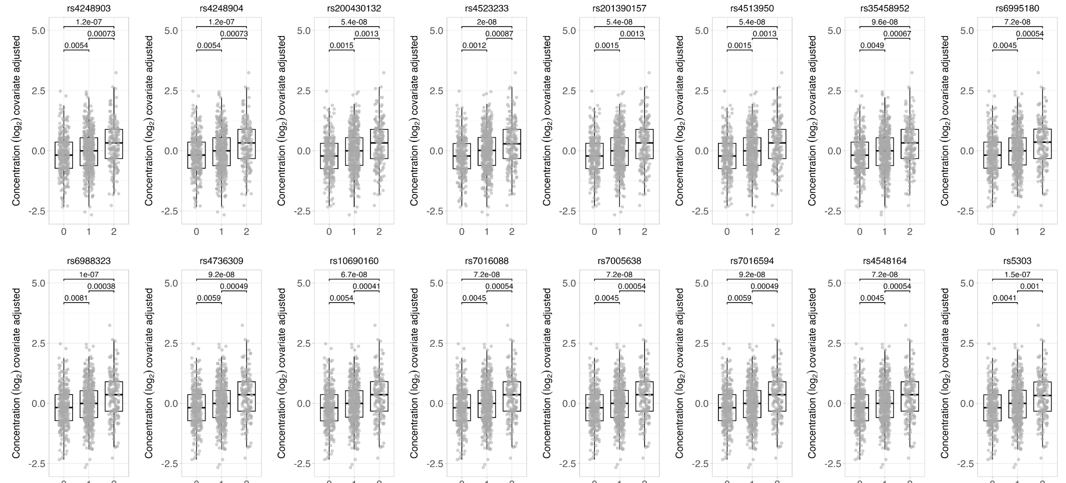

Supplementary Figure 6: SNPs associated with etiocholanolone and 11-deoxycortisol levels in all donors. Box plots of (**A**) etiocholanolone and (**B**) 11-deoxycortisol levels in all donors for significant SNPs identified in the GWAS. Data has been corrected for covariates included in the GWAS (age, BMI, smoking status, as well as PC1 and PC2 of a genetic PCA, menopausal status, HRT, oral contraceptive use, IUD, and tubal ligation). P values displayed here are from a Dunn's test with an FDR adjustment. (n=949 donors)

Supplementary Figure 7:

A DHT

B Testosterone

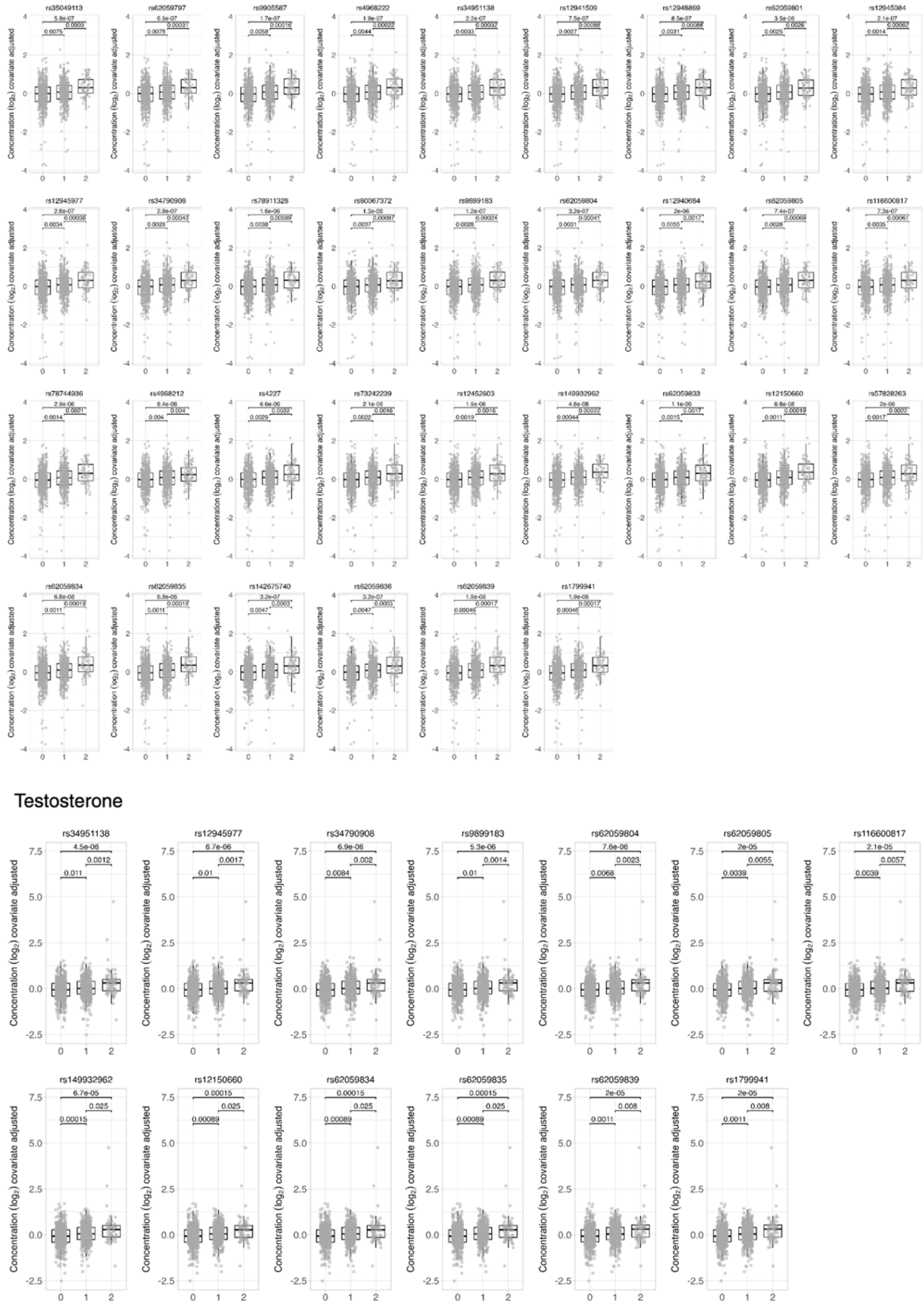

Supplementary Figure 7: SNPs associated with DHT and testosterone levels in all donors. Box plots of (A) DHT and (B) testosterone levels in all donors for significant SNPs identified in the GWAS. Data has been corrected for covariates included in the GWAS (age, BMI, smoking status, as well as PC1 and PC2 of a genetic PCA, menopausal status, HRT, oral contraceptive use, IUD, and tubal ligation). P values displayed here are from a Dunn's test with an FDR adjustment. (n=949 donors)

Supplementary Figure 8

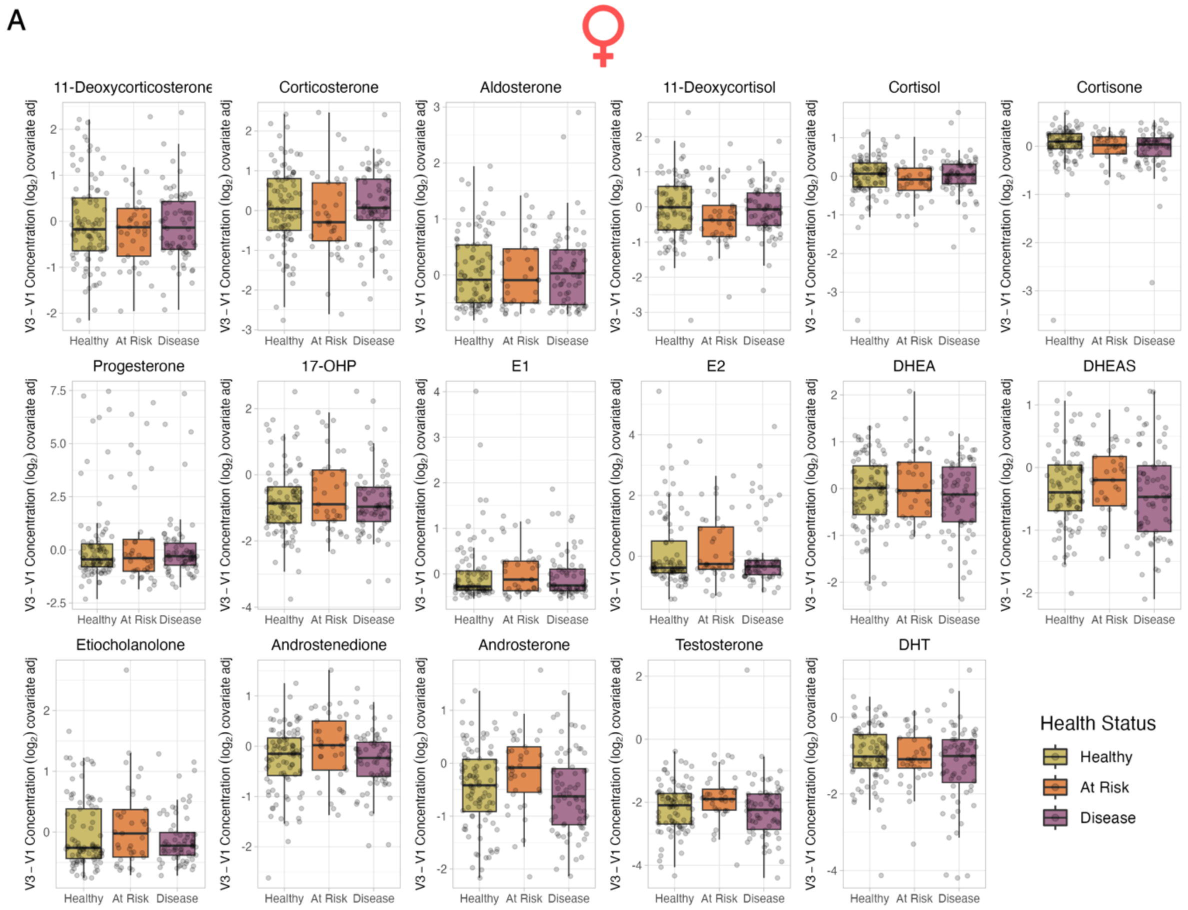

Supplementary Figure 8: Steroid levels are not significantly associated with disease state in female donors. (**A**) Box plots show the individual changes in covariate-corrected steroid hormone levels from V1 to V3 by health status in female donors. There were no significant *q*-values from Mann-Whitney U tests comparing at-risk and disease groups to the healthy group, with correction across all tests. (n=209)

The Milieu Intérieur Consortium¶ is composed of the following team leaders: Laurent Abel, Hôpital Necker, Andres Alcover, Institut Pasteur, Paris, Hugues Aschard, Institut Pasteur, Paris, Philippe Bousso, Institut Pasteur, Paris, Nollaig Bourke, Trinity College Dublin, Petter Brodin, Karolinska Institutet, Pierre Bruhns, Institut Pasteur, Paris, Nadine Cerf-Bensussan, INSERM UMR 1163 – Institut Imagine, Ana Cumano, Institut Pasteur, Paris, Caroline Demangel, Institut Pasteur, Paris, Christophe D'Enfert, Institut Pasteur, Paris, Ludovic Deriano, Institut Pasteur, Paris, Marie-Agnès Dillies, Institut Pasteur, Paris, James Di Santo, Institut Pasteur, Paris, Gérard Eberl, Institut Pasteur, Paris, Jost Enninga, Institut Pasteur, Paris, Jacques Fellay EPFL, Lausanne, Ivo Gomperts-Boneca, Institut Pasteur, Paris, Milena Hasan, Institut Pasteur, Paris, Gunilla Karlsson Hedestam, Karolinska Institutet, Serge Hercberg, Université Paris 13, Molly A Ingersoll, Institut Cochin and Institut Pasteur, Paris, Olivier Lantz, Institut Curie, Rose Anne Kenny, Trinity College Dublin, Mickaël Ménager, INSERM UMR 1163 – Institut Imagine, Frédérique Michel, Institut Pasteur, Paris, Hugo Mouquet, Institut Pasteur, Paris, Cliona O'Farrelly, Trinity College Dublin, Etienne Patin, Institut Pasteur, Paris, Antonio Rausell, INSERM UMR 1163 – Institut Imagine, Frédéric Rieux-Laucat, INSERM UMR 1163 – Institut Imagine, Lars Rogge, Institut Pasteur, Paris, Magnus Fontes, Institut Roche, Anavaj Sakuntabhai, Institut Pasteur, Paris, Olivier Schwartz, Institut Pasteur, Paris, Benno Schwikowski, Institut Pasteur, Paris, Spencer Shorte, Institut Pasteur, Paris, Frédéric Tangy, Institut Pasteur, Paris, Antoine Toubert, Hôpital Saint-Louis, Mathilde Touvier, Université Paris 13, Marie-Noëlle Ungeheuer, Institut Pasteur, Paris, Christophe Zimmer, Institut Pasteur, Paris, Matthew L. Albert, Octant Biosciences, Darragh Duffy§, Institut Pasteur, Paris, Lluis Quintana-Murci, Institut Pasteur, Paris

§ co-coordinators of the Milieu Intérieur Consortium

Additional information can be found at:

https://www.milieuinterieur.fr/en/
